# Designing the response-spectra of microwave metasurfaces: theory and experiments

**DOI:** 10.1515/nanoph-2025-0113

**Published:** 2025-06-17

**Authors:** Yixiang Xu, Yufei Song, Han Zhu, Yifei Wang, Qiong He, Zhuo Wang, Lei Zhou

**Affiliations:** State Key Laboratory of Surface Physics, Key Laboratory of Micro and Nano Photonic Structures (Ministry of Education) and Department of Physics, Fudan University, Shanghai 200438, China; Shanghai Key Laboratory of Metasurfaces for Light Manipulation, Fudan University, Shanghai 200433, China; College of Future Information Technology, Fudan University, Shanghai, 200438, China; Shanghai Frontiers Science Research Base of Intelligent Optoelectronics and Perception, Institute of Optoelectronics, Fudan University, Shanghai 200433, China

**Keywords:** plasmonic resonators, surface current, optical lineshapes, near-field coupling, metasurface

## Abstract

Metasurfaces composed by arrays of coupled plasmonic resonators have attracted tremendous attention due to their extraordinary abilities to manipulate electromagnetic (EM) waves. However, existing theories for such systems are either *empirical* with model parameters obtained by fitting with simulations, or can only be applied to high-frequency systems where metals exhibit *finite* permittivity. Here, we extend our recently established leaky-eigenmode (LEM) theory to the microwave regime where metals exhibit *infinite* permittivity, with all parameters directly computable *without* fitting procedures. After validating our theory with both simulations and experiments on a benchmark metasurface, we illustrate how to utilize the theory to guide designing microwave metasurfaces with freely tailored line-shapes, including particularly the generation of a bound state in the continuum. All theoretical predictions are verified by experiments and simulations. Our study provides a powerful tool to guide designing functional microwave meta-devices for various applications.

## 1 Introduction

**Figure 1:**
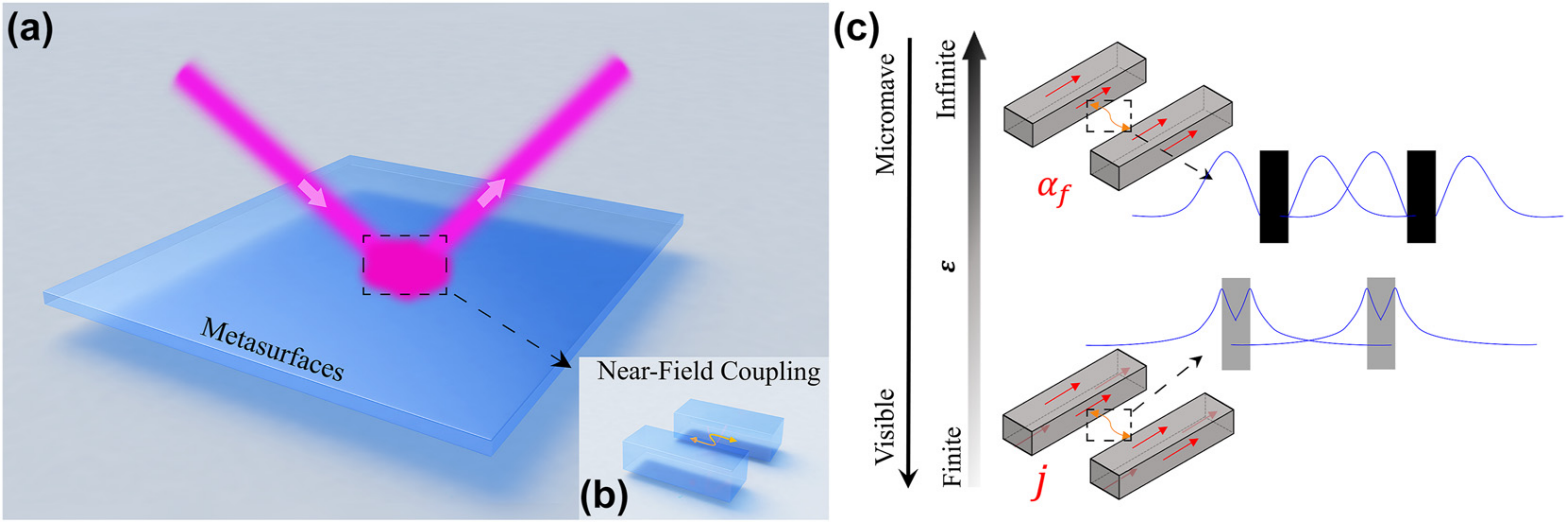
Coupled meta-atoms in metasurfaces: near-field coupling and permittivity properties across visible and microwave regimes. (a) Metasurfaces with complex meta-atoms composed by multiple metallic resonanors where (b) the near-field coupling between inter-resonators playing important role on its optical response. (c) Schematics of permittivity property of metallic resonator at visible and microwave regime where the near-field behavior are determined by body currents ***j*** and surface currents ***α***_***f***_, respectively. The top and bottom insets on the right side of Figure 1c illustrate the different near-field distribution between two resonators at low-frequency and high-frequency, respectively.

Manipulating electromagnetic (EM) waves at will is highly desired in information science and applications. However, EM devices made by conventional dielectrics are too bulky in sizes and exhibit limited wave-control functionalities, as they typically rely on propagating phases of EM waves traveling inside the devices. Metasurfaces [1], [2], [3], ultrathin metamaterials constructed by arrays of subwavelength planar microstructures (e.g., “meta-atoms”) with tailored EM responses, offer an ultra-thin and powerful platform to control EM waves. Many fascinating wave-manipulation effects were demonstrated based on metasurfaces in different frequency domains [4], [5], [6], [7], [8], [9], [10], [11], [12], [13]. Metasurfaces composed by meta-units containing multiple *coupled* resonators (see Figure 1a and b) have attracted much attention recently, since *couplings* among constitutional resonators offer more degrees of freedom to control EM waves [14], [15], [16], [17], [18]. For example, via tailoring the “couplings” between meta-atoms, we can design metasurfaces exhibiting unusual optical line-shapes such as Rabi splitting [19], [20], [21], Fano resonances [22], [23], [24], bound states in continuum (BIC) [25], [26], [27], [28], [29], [30], [31], and so on [32], [33], [34].

Despite of many fascinating effects discovered, however, theoretical tools to guide designing such metasurfaces are insufficiently developed. Full-wave simulations, though being accurate in reproducing experimental results, offer less insights to help understand the intrinsic physics. While theoretical models, including the coupled-mode theory (CMT) [35], [36], [37], [38], Fano’s formula [15], [16] and effective circuit models [17], [18], are intuitive to help understand the intriguing phenomena discovered, they unfortunately require parameter-fitting procedures and thus cannot predict unknown phenomena before numerical simulations are performed. Very recently, based on leaky-eigenmode (LEM) expansions, we established a rigorous theoretical framework to predict the optical response spectra of arbitrarily coupled systems [18], with all involved parameters computed directly without fitting procedures. Assisted with the LEM theory, we can design coupled plasmonic metasurfaces with appropriate *inter-resonator couplings* so as to exhibit desired optical line-shapes, including BIC [39], achromatic no-reflection [17], and freely tailored angular dispersions [40].

However, the LEM theory cannot be directly applied to study microwave metasurfaces. In the LEM theory, all parameters involved (e.g., radiation damping rates, near-field couplings, etc.) are defined as integrations of products between **E**-fields and/or electric currents **j** inside resonators. At high frequencies (i.e., visible or infra-red), metals exhibit finite permittivity *ɛ* so that both **E** and **j** are finite. As the result, all parameters defined in the LEM theory can be unambiguously computed (see Figure 1c). At low frequencies (i.e., the GHz regime) where metals exhibit *infinite* *ɛ*, model parameters defined in the LEM theory cannot be unambiguously computed due to singular distributions of both **E** and **j**. Given so many important applications of microwave metasurfaces, it is highly desired to establish a theory which can be utilized to predict the optical responses of such systems.

Here, we extend the LEM theory to low-frequency regimes and apply it to guide designing coupled microwave metasurfaces exhibiting pre-designed optical line-shapes. The key step is to replace all volume integrations involving **E** and **j** inside metals by integrations of **E** field and surface currents on the resonator surfaces. After validating our theory by both full-wave simulations and experiments on a realistic coupled microwave metasurface, we then illustrate how to utilize our modified LEM theory to guide designing microwave metasurfaces exhibiting freely tailored line-shapes. All theoretical predictions are verified by experiments and full-wave simulations.

## 2 Results

### 2.1 Extended LEM theory for microwave metasurfaces

We start from introducing the key idea of our LEM theory [39], which allows us to study the scattering properties of a photonic system composed of multiple coupled resonators in a host medium. Assuming that each resonator only supports one resonant mode, we first employ analytical or numerical calculations to obtain the LEM wave-functions $\left\{\Psi_{m}^{\text{LEM}}\right\}$ of each individual resonator placed in the host medium, which are the wave-functions computed as each system is *resonantly* excited, but with the illuminating field deducted. We next decompose all LEM wave-functions into near-field (NF) and far-field (FF) parts: $\Psi_{m}^{\text{LEM}}=\Psi_{m}^{\text{NF}}+\Psi_{m}^{\text{FF}}$ . Taking $\left\{\Psi_{m}^{\text{NF}}\right\}$ and the eigenmodes of external ports as NF and FF bases, respectively, we expand the wave dynamical equation and the total wave-function of the coupled system into linear expansions of these wave functions. Using the orthogonal properties of bases functions, we finally derive out the following coupled equations

(1) $$\left\{\begin{array}{c} -i\omega a_{m}=-i\left(\omega_{m}-i\Gamma_{m}\right)a_{m}+\underset{n\neq m}{\sum }\left(-it_{mn}+X_{mn}\right)a_{n}+\underset{q}{\sum }\kappa_{mq}s_{q}^{+}\;\\ s_{q}^{-}=\underset{p}{\sum }s_{p}^{+}c_{qp}+\underset{m}{\sum }a_{m}d_{qm}\; \end{array}\right.$$

to describe the mode dynamics and scattering properties of the coupled system, under external illumination at frequency *ω*. Here, *a*_*m*_ denotes the (complex) amplitude of the mode supported by the *m*-th resonator with its resonant (eigen) frequency and radiation damping rate given by *ω*_*m*_ and Γ_*m*_, respectively, *t*_*mn*_ and *X*_*mn*_ describe the NF and FF couplings between two modes labeled by *m* and *n*, $s_{q}^{+}$ and $s_{q}^{-}$ denote the (complex) amplitudes of the input and outgoing waves traveling along the *q*-th external port, *κ*_*mq*_ and *d*_*qm*_ describe the coupling between the *m*-th resonant mode and the *q*-th port, and *c*_*qp*_ gives the scattering wave to the *q*-th port as the *p*-th port is excited with unit amplitude for the host medium with all resonators taken away.

Although Eq. (1) resemble very much the CMT equations derived previously, the key difference is that all parameters defined here can be unambiguously computed by our NF and FF wave-functions obtained for all resonant modes. Specifically, assuming that our host medium is a non-magnetic medium with permittivity *ɛ*_*h*_ and our resonators are formed by metals with dispersive permittivity $\varepsilon_{m}\left(\omega \right)$ , we have explicitly

(2) $$\left\{\begin{array}{cc} & \Gamma_{m}=\frac{-i\omega_{m}}{2\left\langle \Psi_{m}^{\text{NF}}\left|\right.\Psi_{m}^{\text{NF}}\right\rangle }\int_{V_{m}}\text{d}\tau \left[{\vec{P}}_{m}^{*}\cdot {\vec{E}}_{m}^{\text{FF}}\right]\\ & t_{mn}=\frac{-\omega_{m}}{2\sqrt{\left\langle \Psi_{m}^{\text{NF}}\left|\right.\Psi_{m}^{\text{NF}}\right\rangle *\left\langle \Psi_{n}^{\text{NF}}\left|\right.\Psi_{n}^{\text{NF}}\right\rangle }}\int_{V_{m}}\text{d}\tau \left[{\vec{P}}_{m}^{*}\cdot {\vec{E}}_{n}^{\text{NF}}\right]\\ & X_{mn}=\frac{i\omega_{m}}{2\sqrt{\left\langle \Psi_{m}^{\text{NF}}\left|\right.\Psi_{m}^{\text{NF}}\right\rangle *\left\langle \Psi_{n}^{\text{NF}}\left|\right.\Psi_{n}^{\text{NF}}\right\rangle }}\int_{V_{m}}\text{d}\tau \left[{\vec{P}}_{m}^{*}\cdot {\vec{E}}_{n}^{\text{FF}}\right]\\ & \begin{array}{cc} & \kappa_{mq}=\frac{-\omega_{m}}{2\sqrt{\left\langle \Psi_{m}^{\text{NF}}\left|\right.\Psi_{m}^{\text{NF}}\right\rangle *e}}\int_{V_{m}}\text{d}\tau \left[{\vec{P}}_{m}^{*}\cdot {\vec{E}}_{q}^{+}\right]\\ & d_{qm}=\frac{1}{2\sqrt{\left\langle \Psi_{m}^{\text{NF}}\left|\right.\Psi_{m}^{\text{NF}}\right\rangle *e}}\oint \left[\mu_{0}{\vec{H}}_{q}^{-*}\cdot {\vec{H}}_{m}^{FF}+\varepsilon_{h}{\vec{E}}_{q}^{-*}\cdot {\vec{E}}_{m}^{FF}\right]c\cdot \vec{n}\cdot d\vec{S}\\ & c_{qp}=\frac{1}{2e}\oint \left[\mu_{0}{\vec{H}}_{q}^{-*}\cdot {\vec{H}}_{p}^{+}+\varepsilon_{h}{\vec{E}}_{q}^{-*}\cdot {\vec{E}}_{p}^{+}\right]c\cdot \vec{n}\cdot d\vec{S} \end{array} \end{array}\right.$$

Here, ${\vec{P}}_{m}$ represents the induced polarization intensity inside the *m-*th resonator, ${\vec{E}}_{m}^{\text{NF}}$ and { ${\vec{E}}_{m}^{\text{FF}}$ , $\left.{\vec{H}}_{m}^{\text{FF}}\right\}$ denote, respectively, the NF and FF field distributions associated with the *m*-th resonator, $\left\{{\vec{E}}_{q}^{+},{\vec{H}}_{q}^{+}\right\}$ and $\left\{{\vec{E}}_{q}^{-},{\vec{H}}_{q}^{-}\right\}$ denote, respectively, the EM fields of the incoming and out-going eigenmodes belonging to the *q*-th external port, *c* is the speed of light, $\vec{n}$ denotes the surface normal of the port, and $\left\langle \Psi_{m}^{\text{NF}}\left|\right.\Psi_{m}^{\text{NF}}\right\rangle$ is a normalization constant defined as

(3) $$\left\langle \Psi_{m}^{\text{NF}}\left|\right.\Psi_{m}^{\text{NF}}\right\rangle =\frac{1}{2}\int \text{d}\tau \left[\begin{array}{c} \mu {\vec{H}}_{m}^{\text{NF}*}\cdot {\vec{H}}_{m}^{\text{NF}}+\varepsilon_{\infty }{\vec{E}}_{m}^{\text{NF}*}\cdot {\vec{E}}_{m}^{\text{NF}}\\ +\omega_{0}^{2}{\left(\omega_{p}^{2}\varepsilon_{\infty }\right)}^{-1}{\vec{P}}_{m}^{*}\cdot {\vec{P}}_{m}+{\left(\omega_{p}^{2}\varepsilon_{\infty }\right)}^{-1}{\vec{j}}_{m}^{*}\cdot {\vec{j}}_{m} \end{array}\right]$$

and *e* is another normalization constant which is the energy flux of the incident light multiplied by the area of the port. Here, *μ* is the magnetic permeability of the medium, and we have rewritten the permittivity of both host medium and metal as a unified Lorentz–Drude form: $\varepsilon \left(\omega \right)=\varepsilon_{\infty }+\varepsilon_{0}\frac{\omega_{p}^{2}}{\omega_{0}^{2}-\omega^{2}}$ , where *ɛ*_∞_, *ω*_*p*_ and *ω*_0_ take different values in different regions. Detailed derivations of Eqs. (2) and (3) are provided in Section I of Supplementary Material (SM). Notably, integrations in the first four equations in Eq. (2) are carried out *inside* the *m*-th resonator, whereas those in the reminding two equations are performed on the *surface*s of the external ports.

While the LEM theory was well justified in high-frequency regimes where metals exhibit finite permittivity [38], in the microwave frequency regime, however, the first four parameters defined in Eq. (2) *cannot* be unambiguously computed. In fact, inside the *m*-th resonator, we have ${\vec{P}}_{m}\left(\vec{r}\right)=\left[\varepsilon_{m}\left(\omega_{m}\right)-\varepsilon_{h}\right]{\vec{E}}_{m}^{\text{NF}}\left(\vec{r}\right)$ . Since metals behave as perfect electric conductors (PECs) at microwave frequencies (with *ɛ*_*m*_ → ∞), electric field cannot penetrate inside the metal (i.e., ${\vec{E}}_{m}^{\text{NF}}\left(\vec{r}\right)=0$ inside the *m*-th resonator), making ${\vec{P}}_{m}\left(\vec{r}\right)$ ill-defined. As the results, all *volume* integrations involved in Eq. (2) cannot be unambiguously determined. This also explains why the LEM theory, although being derived from first principles without fitting parameters, has not yet been applied to study microwave metasurfaces.

Here, we solve such an issue by replacing all volume integrations with surface integrations on resonator surfaces. We first replace the polarization density $\vec{P}$ by current density $\vec{j}$ using the relation $\vec{P}=\frac{\vec{j}}{i\omega }$ derived from the charge-conservation law. Next, we note that a realistic metal allows **E**-field (and thus the induced current $\vec{j}$ ) penetrating inside it within a thin layer of skin-depth thickness *δ*. Furthermore, we note that all **E**-fields involved in the first four equations of Eq. (2) (i.e., ${\vec{E}}_{m}^{\text{FF}},{\vec{E}}_{n}^{\text{NF}},{\vec{E}}_{n}^{\text{FF}},{\vec{E}}_{q}^{+}$ ) are *slowly* varying inside the skin-depth region of the *m*-th resonator. Therefore, we can safely replace them by their field values on the surface, and then perform the integrations over $\vec{j}$ along the surface-normal direction (see Section II Figure S1 of SM for more details). Defining the surface-current density as

(4) $${\vec{\alpha }}_{f}\left({\vec{r}}_{\|}\right)=\int_{0}^{\infty }\vec{j}\left(\vec{r}\right)\text{d}r_{⊥}$$

with *r*_⊥_ being the coordinate perpendicular to the surface, we find that the first four equations in Eq. (2) can be rewritten as

(5) $$\left\{\begin{array}{c} \Gamma_{m}=\frac{1}{2\left\langle \Psi_{m}^{\text{NF}}\left|\right.\Psi_{m}^{\text{NF}}\right\rangle }\oint_{S_{m}}\text{d}S\left[{\vec{\alpha }}_{m}^{*}\cdot {\vec{E}}_{m}^{\text{FF}}\right]\\ t_{mn}=\frac{1}{2i\sqrt{\left\langle \Psi_{m}^{\text{NF}}\left|\right.\Psi_{m}^{\text{NF}}\right\rangle *\left\langle \Psi_{n}^{\text{NF}}\left|\right.\Psi_{n}^{\text{NF}}\right\rangle }}\oint_{S_{m}}\text{d}S\left[{\vec{\alpha }}_{m}^{*}\cdot {\vec{E}}_{n}^{\text{NF}}\right]\\ X_{mn}=\frac{-1}{2\sqrt{\left\langle \Psi_{m}^{\text{NF}}\left|\right.\Psi_{m}^{\text{NF}}\right\rangle *\left\langle \Psi_{n}^{\text{NF}}\left|\right.\Psi_{n}^{\text{NF}}\right\rangle }}\oint_{S_{m}}\text{d}S\left[{\vec{\alpha }}_{m}^{*}\cdot {\vec{E}}_{n}^{\text{FF}}\right]\\ \kappa_{mq}=\frac{1}{2i\sqrt{\left\langle \Psi_{m}^{\text{NF}}\left|\right.\Psi_{m}^{\text{NF}}\right\rangle *e}}\oint_{S_{m}}\text{d}S\left[{\vec{\alpha }}_{m}^{*}\cdot {\vec{E}}_{q}^{+}\right] \end{array}\right..$$

On the other hand, the normalization constant defined in Eq. (3) should also be re-evaluated in the microwave regime. Splitting the integration into two parts, one inside the metallic resonators and another outside, we find that the contribution inside the resonators goes to zero in the *ɛ*_*m*_ → −∞ limit (see Section II in SM for derivation details). Therefore, in the microwave regime, we can rewrite Eq. (3) as

(6) $$\left\langle \Psi_{m}^{\text{NF}}\left|\right.\Psi_{m}^{\text{NF}}\right\rangle =\frac{1}{2}\underset{\text{out}}{\int }\text{d}\tau \left[\mu {\vec{H}}_{m}^{\text{NF}*}\cdot {\vec{H}}_{m}^{\text{NF}}+\varepsilon_{h}{\vec{E}}_{m}^{\text{NF}*}\cdot {\vec{E}}_{m}^{\text{NF}}\right]$$

where the integration is only performed outside the metallic resonators (i.e., inside the dielectric medium).

We can employ Eq. (4) to unambiguously determine the four parameters defined our LEM theory, using EM fields computed with metals assumed as PEC in the microwave regime. In particular, surface currents on metallic surfaces can be derived from the calculated **H** fields on surfaces via $\vec{\alpha }=\vec{n}\times \vec{H}$ under the PEC condition. The two remaining parameters defined in Eq. (2) do not involve volume integrations inside metals, and can therefore be calculated using the previously established method [39]. Once all parameters are determined, we can substitute them into Eq. (1) to compute the scattering spectrum of the microwave metasurface under study. However, it is important to note that our approximation will become inadequate when the skin depth of electromagnetic waves is relatively large compared to the wavelength (i.e. when *λ*/*δ* < 40), as is often the case in the optical regime (e.g., >400 THz).

### 2.2 Benchmark test

**Figure 2:**
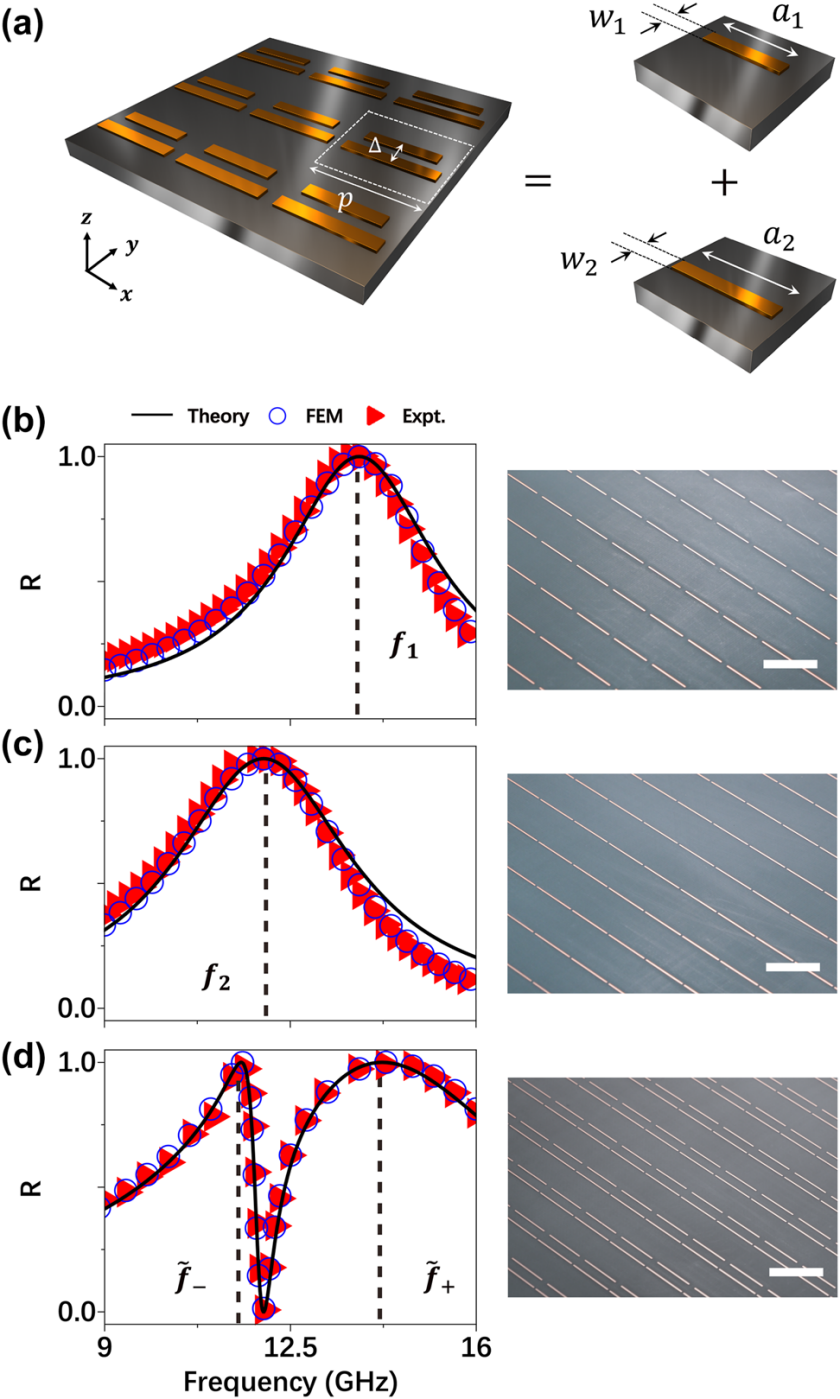
Benchmark test of our LEM theory on a realistic microwave metasurfaces. (a) Schematics of a periodic metasurface with meta-atoms composed by two coupled bar-resonators and placed at the center of its unit-cell. Geometrical parameters: *p* = 10 mm, Δ = 2.6 mm, *a*_1_ = 8.5 mm, *w*_1_ = 0.3 mm, *a*_2_ = 9.5 mm, *w*_2_ = 0.3 mm. And the inter-bar distance is 2.6 mm. All metallic resonators of the fabricated metasurfaces in this paper are made of copper with thickness of 35 um and placed on a dielectric substrate (with *ɛ*_*r*_ = 2.2) with thickness of 1 mm. (b–d) Reflectance spectra of fabricated periodic metasurfaces containing different single bar-resonator (b–c) and two bar-resonators coupled together (d), obtained by experiments (triangles), FEM simulation (circles) and LEM theory (solid line). The right panel of (b–d) are the image of part of the fabricated metasurfaces with scale bar (whith line) of 10 mm.

To validate our modified LEM theory in the microwave regime, we implement it to study a periodic metasurface consisting of meta-atoms constructed by two coupled bar-resonators (see Figure 2a). We first conduct finite-element-method (FEM) simulations to investigate the EM properties of two model systems, each consisting of an array of single-type bar-resonators arranged on a square lattice with periodicity 10 mm, illuminated by *x*-polarized plane waves. From the simulated reflection spectra (circles) shown in Figure 2b and c, we identify two resonant frequencies *f*_1_ and *f*_2_ for two bar-resonators (see dashed lines in Figure 2b and c) from the peak-reflection positions in the spectra. We then utilize our LEM theory to determine the NF and FF wave functions of two resonators, and put them into Eqs. (5) and (6) to compute the parameters (e.g. Γ_*m*_, *κ*_*mq*_, *d*_*qm*_ and *c*_*qp*_) for each single-resonator model system. With these parameters determined, we employ Eq. (1) to predict the reflection spectra of two model systems, which are shown as solid lines in Figure 2b and c. The LEM-calculated reflection spectra exhibit Lorentz-like line-shapes with reflection peaks at the resonant frequencies, being typical features of single-resonator metasurfaces.

We next use the LEM theory to analyze the reflection spectrum of a coupled system comprising two resonators separated by Δ = 2.6 mm within a unit cell (see Figure 2a). While all single-resonator-related parameters (e.g. Γ_*m*_, *κ*_*mq*_, *d*_*qm*_ and *c*_*qp*_) are unchanged, we need to further consider the inter-resonator couplings both in the NF (i.e., *t*_*mn*_) and FF (i.e., *X*_*mn*_), which can again be unambiguously determined by the LEM theory (Eq. (5)). Substituting these parameters into Eq. (1), we compute the reflection spectra of the coupled metasurfaces, and depict them as solid line in Figure 2d. We find that the coupling between two resonators result in two “dressed” modes at frequencies ${\tilde{f}}_{+}$ and ${\tilde{f}}_{-}$ , marked by two dashed lines in Figure 2d, respectively. We emphasize that all these spectra (solid lines in Figure 2b–d) are directly obtained by our LEM theory, without any fitting procedures.

We perform both microwave experiments and FEM simulations to validate the above theoretical predictions. Three metasurface samples are fabricated according to the specified designs (see right panels in Figure 2b–d for their pictures) and their reflection spectra are measured under normal-incidence of *x*-polarized plane waves. We next perform FEM simulations to study the transmission spectra of these metasurfaces. As shown in Figure 2b–d, both experimental results (triangles) and FEM simulations (circles) are in excellent agreement with the LEM-calculated spectra, which unambiguously validate our LEM theory established. All reflection spectra were measured under a small incident angle (approximately 5) rather than normal incidence, in order to simplify the experimental setup, as described in Section VII of SM.

### 2.3 Anomalous behaviors of inter-resonator coupling at microwave frequencies

**Figure 3:**
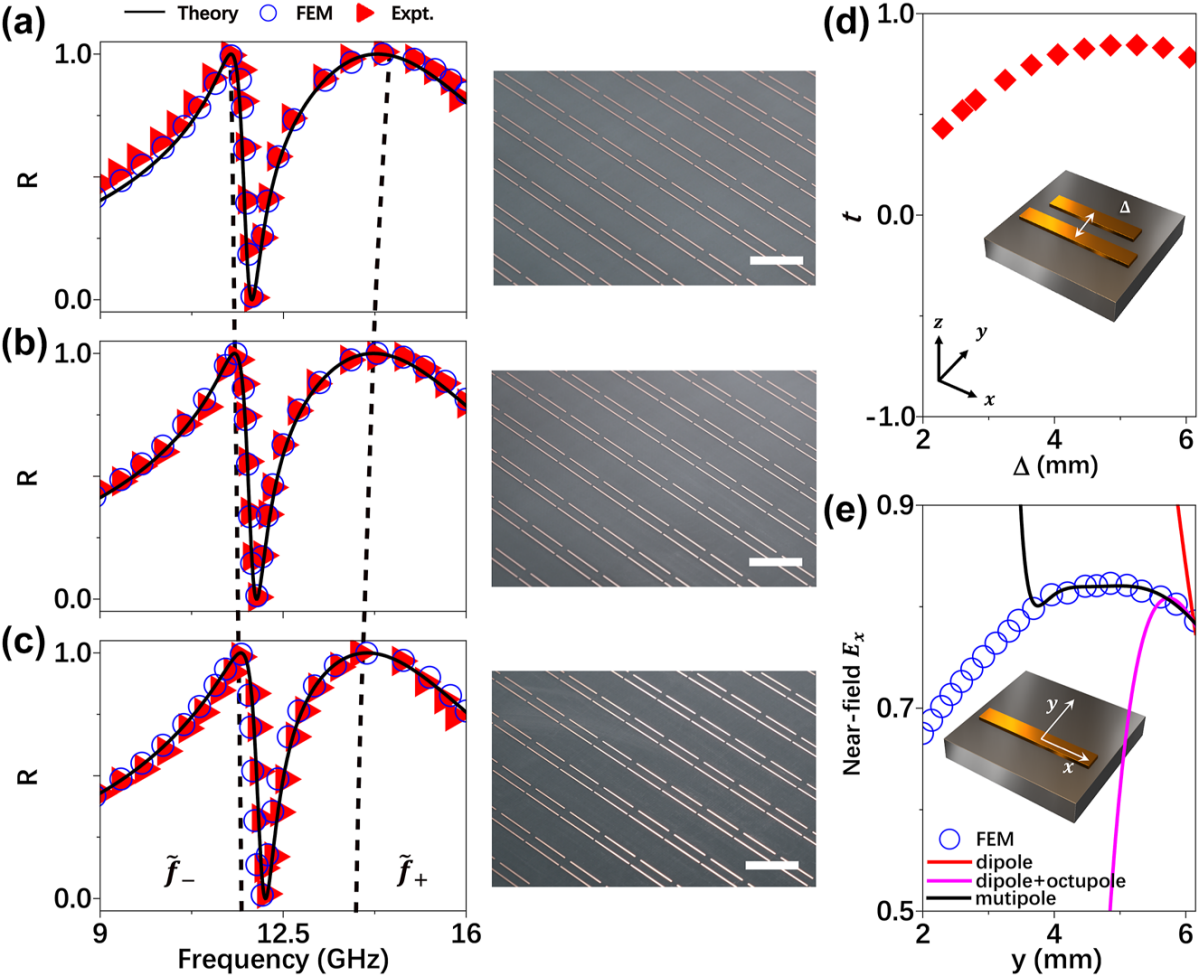
Reflectance spectra and coupling behavior of periodic metasurfaces composed by two coupled bar-resonators with different separation distances. (a–c) Reflectance spectra of fabricated periodic metasurfaces containing two bar-resonators coupled together with different separation distance Δ of 2.8 mm (a), 2.6 mm (b) and 2.3 mm (c), obtained by experiments (triangles), FEM simulation (circles) and theory (solid line), respectively. The dashed lines indicate the resonant frequencies of the “dressed” modes. The right panel of a-c are the pictures of fabricated metasurfaces with scale bar (white line) of 10 mm. (d) Theoritically calculated coupling strength *t* of the two coupled bar-resonators as function of the separation distance Δ. (The geometrical parameters and position of single bar-resonator and coupled bar-resonators are the same as the ones shown Figure 2a). (e) Simulated local E-field amplitude (circles) at differnt position along *y*-axis on the middle line of the single bar-resonantor at the working frequency of 13.8 GHz, with theoritically caltulated E-field amplitude with different multipole modles (solid lines).

We employ the LEM theory to investigate the properties of inter-resonator couplings in such coupled microwave metasurfaces. Keeping the geometric parameters of two single-bar resonators unchanged, we design a series of metasurfaces containing two coupled bar-resonators with continuously varying inter-bar distance Δ. We then employ the LEM theory to study how the NF coupling *t* between two bars varies against Δ and the resulting reflection spectra of different systems. Solid lines in Figure 3a–c are the LEM-computed reflection spectra of three typical systems with Δ being 2.8 mm, 2.6 mm and 2.3 mm, respectively, which are again in perfect agreement with both numerical simulations (circles) and experimental results (triangles) of three fabricated samples (see pictures shown in the right panels of Figure 3a–c). We note that the metasurface studied in Figure 3b is identical to that in Figure 2d. Surprisingly, we notice that the frequency splitting of the two dressed modes ( $\delta \tilde{f}={\tilde{f}}_{+}-{\tilde{f}}_{-}$ ) decreases as Δ changes from 2.8 mm to 2.3 mm, implying that the NF coupling strength *t* is also a decreasing function of inter-resonator distance Δ. This observation initially appears counterintuitive, as typically NF coupling should become stronger as two resonators are closer yielding larger overlapping between two wave-functions [37]. We systematically apply our LEM theory to study more coupled systems with Δ varying in a wide range of [2 mm, 6 mm], and quantify the *t* ∼ Δ relation (solid squares in Figure 3d). Our analysis reveals that *t* exhibits an intriguing non-monotonic dependence on Δ. Specifically, while *t* is an increasing function of Δ in the small-Δ region, it turns to a decreasing function of Δ as Δ is larger than 5 mm.

To understand the underlying physics of such unusual phenomena, we perform FEM simulations to study how the field component $E_{x}^{\text{NF}}$ of a single bar-resonator (with length 9.5 mm) varies along the middle line perpendicular to the bar (see inset to Figure 3e) at its resonant frequency 13.8 GHz. Figure 3e shows that the FEM-simulated $E_{x}^{\text{NF}}$ (blue circles) undergoes an unusual dependence on *y*, similar to the *t* ∼ Δ relation depicted in Figure 3d. We find that such an unusual behavior of NF is dictated by higher order multipoles. Thanks to the symmetry, we only need to consider odd-order multipoles possessed by the bar-resonator. We find that the dipole model can only explain the $E_{x}^{\text{NF}}∼y$ relation in the region of *y* > 6 mm, as shown by the red line in Figure 3e. Meanwhile, as we add octupole to the model and carefully adjust the expansion coefficients of dipole and octupole terms, we find that the applicable region of our model extends to *y* > 5 mm (see the pink line in Figure 3e). Such a trend continues as we add more higher-order multipoles to our model. For example, a model containing five hundred and twelve order multipoles (up to the 2^9^-order term) has been good enough to capture the key features of FEM-computed $E_{x}^{\text{NF}}∼y$ curve in a large region with *y* > 3 mm.

The unusual $E_{x}^{\text{NF}}∼y$ dependence of the bar-resonator can well explain the *t* ∼ Δ relation depicted in Figure 3d, since the most predominant contribution in *t*_*mn*_ is just the ( $\alpha_{m,x}\cdot E_{n,x}^{\text{NF}}$ ) term as shown in Eq. (5). Moreover, results presented here highlight the importance of considering high-order multipoles in modelling EM resonators at GHz frequencies, as compared to their plasmonic counterparts (see more details in Section III of SM). In fact, the PEC property of metal makes a microwave bar-resonator less subwavelength than its plasmonic counterpart and thus high-order multipoles naturally exist in such a microwave resonator. Also, it is much easier to put two resonators closer in the microwave regime than in the high-frequency regimes. These two facts make the contributions from high-order multipoles more significant in the cases that we studied here.

### 2.4 Application of the theory

#### 2.4.1 Predictive generation of BIC

As an application of our LEM theory, we apply it to tailor the optical line-shape of a microwave metasurface composed of an array of meta-atoms consisting of two bar-resonators coupled together. In particular, we illustrate how to design a metasurface exhibiting a BIC in a predictive way. As shown in Figure 3a–c, inter-resonator coupling *t* can significantly alter the line-shape of the coupled metasurface, through manipulating the essential properties of two “dressed” modes including their eigen frequencies and bandwidths. For the present two-mode two-port system, we find that the first equation in Eq. (1) can be explicitly written as

(7) $$\begin{array}{ll} -i\omega \left(\begin{array}{c} a_{1}\\ a_{2} \end{array}\right) & =-i\left(\begin{array}{cc} \omega_{1} & t\\ t & \omega_{2} \end{array}\right)\left(\begin{array}{c} a_{1}\\ a_{2} \end{array}\right)+\left(\begin{array}{cc} -\Gamma_{1} & X\\ X & -\Gamma_{2} \end{array}\right)\left(\begin{array}{c} a_{1}\\ a_{2} \end{array}\right)\\ & \;+\left(\begin{array}{c} \kappa_{11}\\ \kappa_{21} \end{array}\right)s_{1}^{+} \end{array}$$

where $\left\{\omega_{1},\omega_{2}\right\}$ and {Γ_1_, Γ_2_} denote the eigen frequencies and radiation damping rates of two original modes, $\left\{t,X\right\}$ are the NF and FF coupling strengths between two modes, and {*κ*_11_, *κ*_21_} describe the couplings of these two modes with the first external port (reflection side).

Diagonalizing the first matrix at the right-hand side of Eq. (7), we obtain

(8) $$\begin{array}{ll} -i\omega \left(\begin{array}{c} {\tilde{a}}_{+}\\ {\tilde{a}}_{-} \end{array}\right) & =-i\left(\begin{array}{cc} {\tilde{\omega }}_{+} & 0\\ 0 & {\tilde{\omega }}_{-} \end{array}\right)\left(\begin{array}{c} {\tilde{a}}_{+}\\ {\tilde{a}}_{-} \end{array}\right)+\left(\begin{array}{cc} -{\tilde{\Gamma }}_{+} & \tilde{X}\\ \tilde{X} & -{\tilde{\Gamma }}_{-} \end{array}\right)\left(\begin{array}{c} {\tilde{a}}_{1}\\ {\tilde{a}}_{2} \end{array}\right)\\ & \;+\left(\begin{array}{c} {\tilde{\kappa }}_{11}\\ {\tilde{\kappa }}_{21} \end{array}\right)s_{1}^{+} \end{array}$$

where ${\tilde{\Gamma }}_{\pm }=\frac{\Gamma_{1}+\Gamma_{2}}{2}\pm \frac{2t\sqrt{\Gamma_{1}\Gamma_{2}}+\Delta \omega \Delta \Gamma }{2\sqrt{t^{2}+{\left(\Delta \omega \right)}^{2}}}$ and ${\tilde{\omega }}_{\pm }=\frac{\omega_{1}+\omega_{2}}{2}\pm \sqrt{t^{2}+{\left(\Delta \omega \right)}^{2}}$ with $\Delta \omega =\frac{\omega_{1}-\omega_{2}}{2}$ and ΔΓ = Γ_1_ − Γ_2_. More derivation details can be found in Section IV of SM. Defining two new parameters $\Delta \tilde{\omega }={\tilde{\omega }}_{+}-{\tilde{\omega }}_{-}$ and $\Delta \tilde{\Gamma }={\tilde{\Gamma }}_{+}-{\tilde{\Gamma }}_{-}$ , we get that

(9) $$\left\{\begin{array}{c} \Delta \tilde{\omega }=2\sqrt{t^{2}+{\left(\Delta \omega \right)}^{2}}\;\\ \Delta \tilde{\Gamma }=\left(2t+\Delta \omega \Delta \Gamma \right)/\sqrt{t^{2}+{\left(\Delta \omega \right)}^{2}}\; \end{array}\right.$$

Here and in what follows, we have normalized all involved physical quantities (such as $\Delta \tilde{\omega }$ , ${\tilde{\Gamma }}_{\pm }$ , ΔΓ and *t*) by $\sqrt{\Gamma_{1}\Gamma_{2}}$ to make them dimensionless.

**Figure 4:**
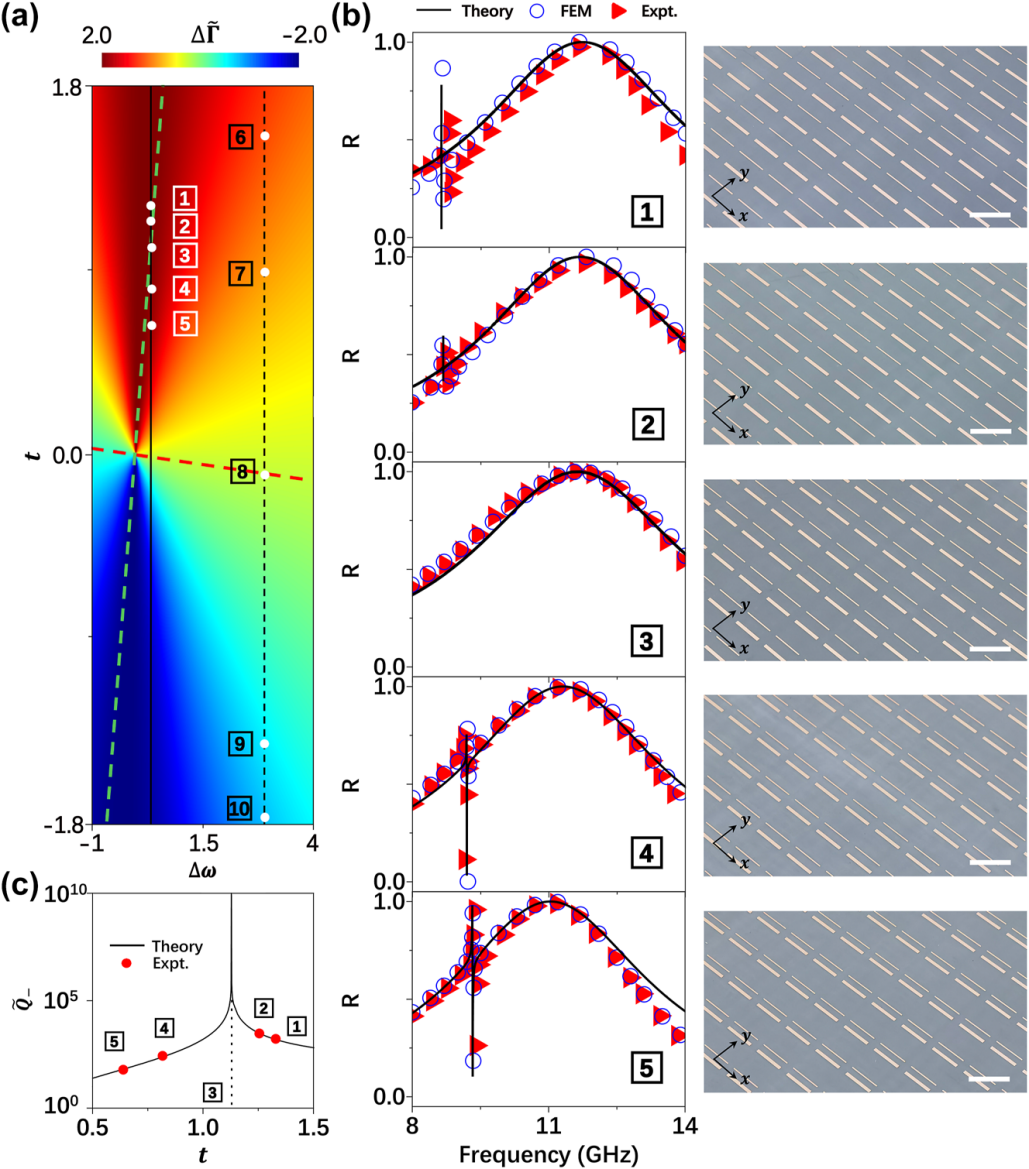
Experimental demonstration for realizing BIC with coupled microwave metasurface. (a) Calculated the difference of radiation damping for two dressed modes $\Delta \tilde{\Gamma }$ (color map) versus *t* and Δ*ω* for metasurfaces containing two resonators coupled together. (b) Reflection spectra of five metasurfaces containing two bar-resonators coupled differently corresponding to the point #1–5 labelled on (a), obtained by our theory (solid lines), FEM simulations (circles) and experiments (triangles). Insets: pictures of fabricated samples with separation 7.4 mm, 6.8 mm, 5.6 mm, 4.4 mm and 3.4 mm between two coupled bar-resonators, respectively. The scale bars (white lines) is 15 mm. Other geometrical parameters: The widths of the metal bars are 1.25 mm and 0.45 mm, and their lengths are 11.5 mm and 12 mm, respectively. (c) Quality factor of the low-frequency dressed mode ${\tilde{Q}}_{-}$ as a function of the inter-resonator coupling *t* obtained by our theory (line), and retrieved from experimental spectra by fitting with CMT (solid circle). The singular behavior is theoretically represented by system #3, which corresponds to the BIC condition with a diverging *Q*-factor, and cannot be experimentally realized within the limitations of our measurement setup.

We now employ Eq. (9) to analytically study how to tailor the line-shapes of coupled microwave metasurfaces. Figure 4a maps the calculated $\Delta \tilde{\Gamma }$ versus detuning Δ*ω* and *t* in the coupled systems. For two original modes (and thus Δ*ω*) given, Eq. (9) indicates that $\Delta \tilde{\Gamma }$ can be significantly modulated via changing the inter-resonator coupling *t*. Meanwhile, we note that the orthogonal transformation does not change the trace of a matrix, and thus ${\tilde{\Gamma }}_{+}+{\tilde{\Gamma }}_{-}$ must keep as a constant along with the variation of *t*, i.e., $\Gamma_{\text{tot}}=\Gamma_{1}+\Gamma_{2}={\tilde{\Gamma }}_{+}+{\tilde{\Gamma }}_{-}$ . Here, we have fixed the total radiation damping of two original modes as Γ_tot_ = 2.0. Therefore, the conditions $\Delta \tilde{\Gamma }=\pm \Gamma_{\text{tot}}$ correspond to the scenarios where one of two “dressed” mode exhibit zero radiation damping, thereby realizing a BIC. The green dashed line in Figure 4a corresponds to the condition $\Delta \tilde{\Gamma }=\pm \Gamma_{\text{tot}}$ , providing a guidance to design appropriate metasurfaces exhibiting a BIC. The red dashed line represents the case ${\tilde{\Gamma }}_{+}={\tilde{\Gamma }}_{-}$ , indicating the systems should contain two equal-bandwidth spectral peaks. Choosing metasurfaces along trajectories intersecting the $\Delta \tilde{\Gamma }=\pm \Gamma_{\text{tot}}$ contour (e.g., the green dashed line in Figure 4a), we expect that the resulting line-shapes must exhibit fascinating evolution, with the very metasurface positioned on the $\Delta \tilde{\Gamma }=\pm \Gamma_{\text{tot}}$ boundary exhibits a BIC.

To illustrate such a line-shape evolution, we purposely choose five systems (marked as #1–5) on the solid black line across the green boundary line $\Delta \tilde{\Gamma }=\pm \Gamma_{\text{tot}}$ in Figure 4a. These systems all exhibit identical Δ*ω* = 0.35 but with different inter-resonator coupling *t*. We find that all these metasurfaces can be realized by the coupled meta-atoms containing two metallic bars designed in Figure 2 (with Δ*ω* = 0.35 and ΔΓ = 0.33), but with appropriate inter-bar distance Δ yielding the desired values of *t* (see Section V of SM). With all these metasurfaces explicitly designed, we employ our LEM theory to compute their reflection spectra based on the single-resonator properties already available (see Figure 2), and depict the obtained spectra as solid lines in Figure 4b. We note that the LEM-predicted reflection spectra of these systems evolve with the same trend as shown in the phase diagram Figure 4a. Specifically, the reflection spectra (solid lines in Figure 4b) evolve from containing two peaks to containing only a single peak (system #3) and then back to containing two peaks again, as *t* decreases. Obviously, metasurface #3 exhibits the desired BIC.

We fabricate out all five samples according to the designs (see the right panel of Figure 4b for their pictures) and employ experiments and numerical simulations to verify the above predictions. Shining the metasurfaces by normally incident *x*-polarized plane waves, we measure their reflection spectra using the same setup as in Figures 2 and 3. As shown in Figure 4b, both experimental (triangles) and simulation (circles) results agree very well with the LEM-calculated ones (solid lines). Specifically, reflection peak of the low-frequency mode does not appear for sample #3 in both simulations and experiments, already signifying the appearance of a BIC in consistency with theoretical prediction.

We explicitly illustrate, both theoretically and experimentally, how the radiative *Q* factor of the low-frequency dressed mode varies against *t*. We depict *Q* values retrieved from five measured transmission spectra as circles in Figure 4c, compared with the solid line representing *Q* values retrieved LEM-computed transmission spectra for a series of coupled metasurfaces with varying *t*. Experimentally measured *Q* factors are in excellent agreement with theoretical predictions, both showing that *Q* diverges at a specific point indicating the emergence of a BIC. To understand the physics behind such a fascinating effect, we employ FEM to simulate the Re(Ex) distributions of waves radiated from three systems, i.e., two metasurfaces containing periodic arrays of two single-type bar resonators and system #3 containing the coupled meta-atoms, under excitations of incident waves at the BIC working frequency. Three computed Re(Ex) patterns are compared in Figure S5 in SM. Clearly, while two single resonators still radiate well to the far-field, the coupled meta-atom does not. In fact, wave-function of the “dressed” mode in the coupled system is a linear combination of two original modes [38], ${\tilde{\Psi }}_{\text{\_}}^{\text{LEM}}=m_{21}\Psi_{1}^{\text{LEM}}+m_{22}\Psi_{2}^{\text{LEM}}$ , with *m*_21_ and *m*_22_ being two coefficients dictated by the coupling *t*. Therefore, tuning the value of *t* can dramatically alter the coefficients *m*_21_ and *m*_22_ (see more details in Section IV of SM), thereby modifying the total radiation of the “dressed” mode. For sample #3 with *t* = 1.13, we find *m*_21_ = −0.65 and *m*_22_ = 0.76, making radiations from two original modes completely cancel each other, leading to the BIC (see Figure S5 in Section V of SM). Although BICs have been widely studied in different frequency domains, previously realized BIC systems were mostly dielectric ones. While dielectric metasurfaces suffer less from absorption losses, they unfortunately require relatively large thicknesses and thus are not as compact as their plasmonic counterparts. While metals exhibit relatively high losses in optical domain, they behave as PEC in the microwave regime and thus can be suitable materials to construct BIC systems. Therefore, our LEM theory provides a usual guide to design ultra-thin loss-free BIC systems in the microwave regime, which can find many applications.

It is worth mentioning that our extended LEM method can not only be used to study the near-field coupling effects induced by lateral shifts between meta-atoms, but also to understand the influence of near-field coupling and far-field radiation behavior caused by vertical shifts, as demonstrated in Ref. [17] in the optical regime. In addition, our LEM theory is applicable under oblique incidence (see more details in Section VIII in SM), provided that the subwavelength characteristics of the metasurfaces are maintained across different incident angles.

#### 2.4.2 Tailoring the optical line-shape

**Figure 5:**
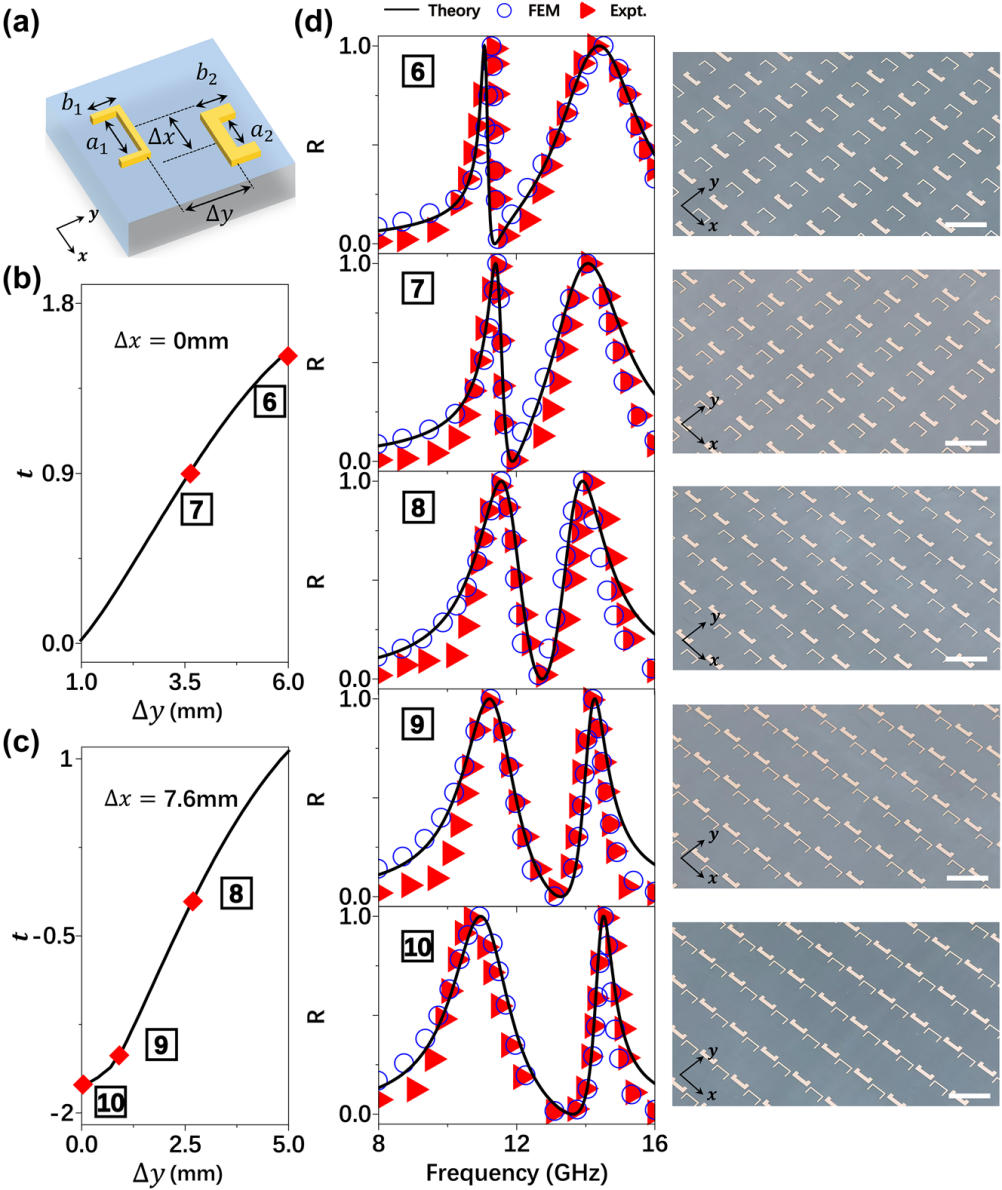
Experimental demonstration for line-shape tailoring: (a) schematics of a unitcell in a periodic metasurface composed by two coupled resonators. Geometrical parameters: *a*_1_ = 5.08 mm, *b*_1_ = 2.93 mm, *a*_2_ = 3.5 mm, *b*_2_ = 2.15 mm and their linewidths are 0.4 mm and 1.3 mm, respectively with period is 15 mm. (b–c) The relationship between the relative position variation of the resonators at points #6–#10 and the near-field coupling strength *t*. (d) Optical line-shapes (reflection spectra) of the metasurfaces containing two-resonators coupled differently with different Δ corresponding to the 5 points labelled as #6–10 obtained by our theory (solid lines), FEM simulations (circles), experiments (triangles). Insets: pictures of fabricated samples with the scale bars (white lines) is 15 mm, other parameters can be found in Table S2 in SM.

Apart from the predictive generation of a BIC, we further illustrate how to employ our theory to freely tailor the optical line-shape of the coupled system. As shown in the phase diagram Figure 4a, radiation-damping difference $\Delta \tilde{\Gamma }$ can transit across different boundary lines by varying the coupling *t*, indicating that the bandwidths of two peaks in reflection spectra the can be dramatically modulated. To illustrate such modulation effect more clearly, we choose another 5 systems along the dashed black line with fixed Δ*ω* = 3.04 in Figure 4a (marked as #6–10), and employ our LEM theory to calculate their reflection spectra. As shown by solid lines in Figure 5d, we find that decreasing the coupling strength *t* can increase (decrease) the bandwidth of the low (high) – frequency dressed mode, thereby significantly altering the optical line-shape of the coupled metasurface. In particular, for system #8 which just sits on the boundary line with $\Delta \tilde{\Gamma }=0$ , its reflection spectrum contains two peaks with equal bandwidth (see #8 solid line in Figure 5d), as expected.

We now experimentally verify the above predictions. We first design two new resonators with appropriate $\left\{\omega_{i},\Gamma_{i}\right\}$ according to the requirement ( $\frac{\Delta \omega }{\sqrt{\Gamma_{1}\Gamma_{2}}}=3.04\left.\right)$ (see Section VI of SM for more design details). Figure 5a depicts the geometric structures of two designed resonators. We next determine the relative configurations between two resonators to yield the desired values of coupling *t*. As shown in Figure 5a–c, changing the relative position Δ*x* and Δ*y* between two resonators dramatically change the value of *t*. According to Figure 5b and c, we choose five different relative configurations yielding appropriate *t* as required by those circles labeled by #6–10 shown in Figure 4a, and then design our samples #6–10 accordingly. We fabricate them out according to the designs (see the right panel of Figure 5d for their pictures), and experimentally measure their transmission spectra under illuminations of *x*-polarized incident plane waves. We also perform FEM simulations to calculate the transmission spectra of these metasurfaces. As shown in Figure 5d, experimental (triangles) and simulation results (circle) are in nice agreement with theoretical predictions, demonstrating the powerfulness of our LEM theory in predicting the optical line-shape of a coupled metasurface. The observed high-frequency discrepancy originates from our theoretical framework’s intentional exclusion of secondary modes beyond 16 GHz, a necessary simplification maintaining physical interpretability of the dominant mode-coupling mechanics below 15 GHz.

## 3 Conclusions

In summary, we have extended our previously established LEM theory to low-frequency regimes where metals exhibit infinite permittivity, by replacing all volume integrations involving **E** and **j** by surface integrations on the resonator surfaces. After validating our extended LEM theory by both simulations and experiments, we revealed the underlying physics of the abnormal behaviors of near-field couplings at microwave frequencies, and applied the theory to guide designing coupled metasurfaces with tailored line-shapes. Specifically, we illustrate how to design a microwave metasurface exhibiting a BIC, and how to design coupled metasurfaces with freely tailored optical line-shapes. All theoretical predictions are verified by microwave experiments and full-wave simulations. Our study provides a powerful tool to guide designing functional microwave meta-devices for various applications, such as smart radar systems requiring real-time reconfigurable filters for microwave communications, lab-on-chip biosensors leveraging BIC-engineered linewidth compression for molecular fingerprinting.

## Supplementary Material

Supplementary Material Details
