# Supplementary material for: Designing the response-spectra of microwave metasurfaces: theory and experiments
Source: Nanophotonics. 2025 Jun 17;14(23):4229–39. doi: 10.1515/nanoph-2025-0113 (PMC12617734; doi:10.1515/nanoph-2025-0113)
Supplement: Supplementary file 1 — Supplementary Material Details [file j_nanoph-2025-0113_suppl_001.pdf]

*Supplementary information for*

# Designing the response-spectra of microwave metasurfaces: theory and experiments

**Yixiang Xu[1†], Yufei Song[1†], Han Zhu[1†], Yifei Wang, Qiong He[1, 2,*], Zhuo Wang[1,2,3*]and Lei Zhou[1, 2,*]**

[1] State Key Laboratory of Surface Physics, Key Laboratory of Micro and Nano Photonic Structures (Ministry of Education) and Physics Department, Fudan University, Shanghai, China, 200433

[2] Shanghai Key Laboratory of Metasurfaces for Light Manipulation, Fudan University, Shanghai 200433, China

[3] Shanghai Frontiers Science Research Base of Intelligent Optoelectronics and Perception, Institute of Optoelectronics, Fudan University, Shanghai 200433, China.

[*]**Corresponding author: Qiong He,** E-mail: Qionghe@fudan.edu.cn
**Zhuo wang,** E-mail: zhuowang15@fudan.edu.cn
**Lei Zhou**, E-mail:phzhou@fudan.edu.cn

†These authors contributed equally to this work.

**List of contents:**

**Section I – Introduction about LEM and Detailed Derivations of Eq. (2-3)**

**Section II – Explanation of Eq. (4) and Re-derivation of $\langle \Psi_m^{\mathrm{NF}} | \Psi_m^{\mathrm{NF}} \rangle$ for microwave frequency regime**

**Section III – $E_x^{\mathrm{NF}}$ distribution of plasmonic resonators at optical frequency**

**Section IV – Derivation of Eq. (8)**

**Section V – Physical mechanism of the BIC for the system #3 in Fig. 4**

**Section VI – Details information about single resonators in Figs. 4 and 5**

**Section VII – Reflection spectrum experimental system**

**Section VIII – Applicability of LEM theory for oblique incident case**

## Section I – Introduction about LEM and Detailed Derivations of Eq. (2-3)

As for a generic coupled open system, we examine the scattering phenomena of a system composed of $m$ arbitrary resonators positioned at various locations within a host medium, subjected to specific external illumination. The region housing resonators is intricately linked to the external continuum through n ports, each characterized by distinct properties. And then we are required to solve the following Schrödinger-like equation:

$$\hat{H}\Psi(\vec{r},\omega) = \omega\Psi(\vec{r},\omega) \tag{S1.1}$$

where $\Psi(\vec{r},\omega)$ is the total wave function, and $\hat{\mathrm{H}} = \hat{\mathrm{H}}_{\mathrm{h}} + \sum_m \hat{\mathrm{V}}_m$ is the Hamiltonian of the whole system with $\hat{\mathrm{H}}_{\mathrm{h}}$ describing the host medium and the $\hat{\mathrm{V}}_m$ potential contributed by the $m$th resonator.

Here, we define a set of wave functions$\{\Psi_m^{\mathrm{LEM}}(\vec{r},\omega_m), m = 1, \dots, M\}$, which are the approximate solutions of the Hamiltonian $\hat{\mathrm{H}}_m = \hat{\mathrm{H}}_{\mathrm{h}} + \hat{\mathrm{V}}_m$, describing the subsystem containing only the $m$th resonator. And we can employ the following approach to obtain $\Psi_m^{\mathrm{LEM}}(\vec{r},\omega)$. We can solve $\hat{\mathrm{H}}_m\Psi_m = \omega\Psi_m$ to obtain $\Psi_m$ either analytically or numerically, and subsequently derive the response spectrum of the system.

Next, we pinpoint the resonance frequency, denoted as $\omega_m$, of the $m$th resonator by locating the peak in the response spectrum. By selecting a background that characterizes the system at a frequency significantly distant from any resonances, we can derive the background wave function $\Psi_{\mathrm{B}}$ through the illumination of the background medium with identical external light. And we can get the LEM wave function through $\Psi_m^{\mathrm{LEM}} = \Psi_m - \Psi_{\mathrm{B}}$ for the $m$th resonator. According to leaky eigenmodes (LEM)[1], $\Psi_m^{\mathrm{LEM}}(\vec{r},\omega_m)$ contains both NF and FF part:

$$\Psi_m^{LEM}(\vec{r},\omega_m) = \Psi_m^{NF}(\vec{r},\omega_m) + \Psi_m^{FF}(\vec{r},\omega_m) \tag{S1.2}$$

Where $\Psi_m^{\mathrm{NF}}$ and $\Psi_m^{\mathrm{FF}}$ represent the NF and FF parts of the wave function respectively. Generally speaking, for any given system with clearly defined external ports, we can consistently project $\Psi_m^{\mathrm{LEM}}$ onto the port modes on reference planes of all external ports. Then, we can construct $\Psi_m^{\mathrm{FF}}$ using these port modes, which are assumed to fill the entire space. With $\Psi_m^{\mathrm{FF}}$ determined, we can then obtain $\Psi_m^{\mathrm{NF}}$ numerically according to Eq. (S1.2).

Finally, we can construct the total wave function[1] as

$$\Psi(\vec{r},\omega) = \sum_q s_q^+ \Psi_B^q + \sum_n a_n |\Psi_n^{LEM}\rangle \tag{S1.3}$$

Where $\{a_n\}$ represents a set of unknown coefficients representing the strengths of fields scattered by different resonators under external illumination represented by $\{s_q^+\}$ denoting the excitation amplitudes at different incoming ports.

And $\Psi_{\mathrm{B}}^{q}$ denotes the background wave function obtained when only the $q$th port is excited with unit amplitude. By substituting Eq. (S1.3) into Eq. (S1.1) and projecting both sides with $\langle\Psi_{n}^{\mathrm{NF}}|$, then we can get the following equations to determine $\{a_n\}$:

$$-i\omega a_m = -\mathrm{i}(\omega_{\mathrm{m}} - i\Gamma_m)a_m + \sum_{n\neq m}(-it_{mn} + X_{mn})a_n + \sum_q \kappa_{mq}s_q^{+} \tag{S1.4}$$

Next, we multiply both sides of $\Psi(\vec{r},\omega)$ defined in Eq. (S1.3) by each FF outgoing basis $\langle k_q^{-}|$ (LSA), and then perform the field integrations at the reference planes of all ports. We finally can obtain the following equations:

$$s_q^{-} = \sum_p s_p^{+}\, c_{qp} + \sum_m a_m d_{qm} \tag{S1.5}$$

Which describes the strengths of scattered fields measured at different external ports.

Now, let us see our theory in photonic systems, which is described inhomogeneous permittivity function $\varepsilon(\vec{r},\omega)$ in which at each local point $\vec{r}$, the permittivity is $\varepsilon(\omega) = \varepsilon_{\infty}[1 + \frac{\omega_p^2}{\omega_0^2-\omega^2+i\omega\Gamma_e}]$,where $\varepsilon_{\infty}, \omega_0, \omega_p$ and $\Gamma_{\mathrm{e}}$ are all position- and frequency-independent parameters, describing the local properties of constituent materials. The governing equations (i.e., Maxwell's equations in the frequency domain) can be formally rewritten as Eq. (S1.1) [2], here the Hamiltonian is given by

$$\hat{H} = \begin{pmatrix} 0 & -\frac{i}{\mu}\nabla\times & 0 & 0 \\ \frac{i}{\varepsilon_{\infty}}\nabla\times & 0 & 0 & \frac{i}{\varepsilon_{\infty}} \\ 0 & 0 & 0 & i \\ 0 & i\omega_p^2\varepsilon_{\infty} & -i\omega_0^2 & -i\Gamma_e \end{pmatrix} \tag{S1.6}$$

And the wave function is defined as $\Psi(\vec{r}) = (\vec{H}\ \vec{E}\ \vec{P}\ \vec{J})$, where $\vec{E}, \vec{H},$ and $\vec{P}$ denoting the electric, magnetic, and polarization fields, respectively, and $\vec{J} = \frac{d\vec{P}}{t}$ describing the polarization current.

According to the Hamiltonian form of photonic systems (Eq. (S1.6)), we find that the potential operator contributed by the m-th scatter as shown in Eq. (S1.7).

$$\hat{\mathrm{V}}_m = \hat{\mathrm{H}}_m - \hat{\mathrm{H}}_h = \begin{pmatrix} 0 & -i\left(\frac{1}{\mu_m}-\frac{1}{\mu_h}\right)\nabla\times & 0 & 0 \\ i\left(\frac{1}{\varepsilon_{\infty,m}}-\frac{1}{\varepsilon_{\infty,h}}\right)\nabla\times & 0 & 0 & -i\left(\frac{1}{\varepsilon_{\infty,m}}-\frac{1}{\varepsilon_{\infty,h}}\right) \\ 0 & 0 & 0 & 0 \\ 0 & i\left(\omega_{p,m}^2\varepsilon_{\infty,m} - \omega_{p,h}^2\varepsilon_{\infty,h}\right) & -i\left(\omega_{0,m}^2 - \omega_{0,h}^2\right) & 0 \end{pmatrix} \tag{S1.7}$$

Where $\varepsilon_{\infty,m}$, $\mu_m$ and $\omega_{0,m}$ are position-dependent functions describing the properties of the m-th scatter, and $\varepsilon_{\infty,h}$, $\mu_h$, and $\omega_{0,h}$ describe the homogeneous host medium. We consider the lossless case (i.e., $\Gamma^{a} = 0$), and consider a special case that scatters formed by plasmonic metals (Au or Cu) and the

host medium is just air, then we have $\varepsilon_{\infty,m} = \varepsilon_{\infty,h} = \varepsilon_0$, $\mu_m = \mu_h = \mu_0$ and thus Eq. (S1.7) can be simplified as

$$\hat{V}_m = \begin{pmatrix} 0 & 0 & 0 & 0 \\ 0 & 0 & 0 & 0 \\ 0 & 0 & 0 & 0 \\ 0 & i\omega_{p,m}^2\varepsilon_0 & 0 & 0 \end{pmatrix} \tag{S1.8}$$

The parameters in Eqs. (S1.4) and (S1.8) are clearly defined and can be computed using the provided integrals. These integrals offer a straightforward method to determine the values of the parameters, ensuring clarity and precision in their calculation.

$$\begin{cases} \Gamma_m = i\langle\psi_m^{\mathrm{NF}}|\hat{\mathrm{V}}_m|\psi_m^{\mathrm{FF}}\rangle_V \\ t_{mn} = \langle\psi_m^{\mathrm{NF}}|\hat{\mathrm{V}}_m|\psi_n^{\mathrm{NF}}\rangle_V \\ X_{mn} = -i\langle\psi_m^{\mathrm{NF}}|\hat{\mathrm{V}}_m|\psi_n^{\mathrm{FF}}\rangle_V \\ \kappa_{mq} = -i\langle\psi_m^{\mathrm{NF}}|\hat{\mathrm{V}}_m|\Psi_{\mathrm{B}}^{q}\rangle_V \\ c_{qp} = \langle k_q^-|\Psi_{\mathrm{B}}^{p}\rangle_S \\ d_{qm} = \langle k_q^-|\psi_m^{\mathrm{FF}}\rangle_S \end{cases} \tag{S1.9}$$

Where "*V*" and "*S*" denote whether the integrals are performed over the entire volume or at the reference plane of a port.

**Section II –Explanation of Eq. (4) and Re-derivation of $\langle \Psi_m^{\mathrm{NF}} | \Psi_m^{\mathrm{NF}} \rangle$ for microwave frequency regime**

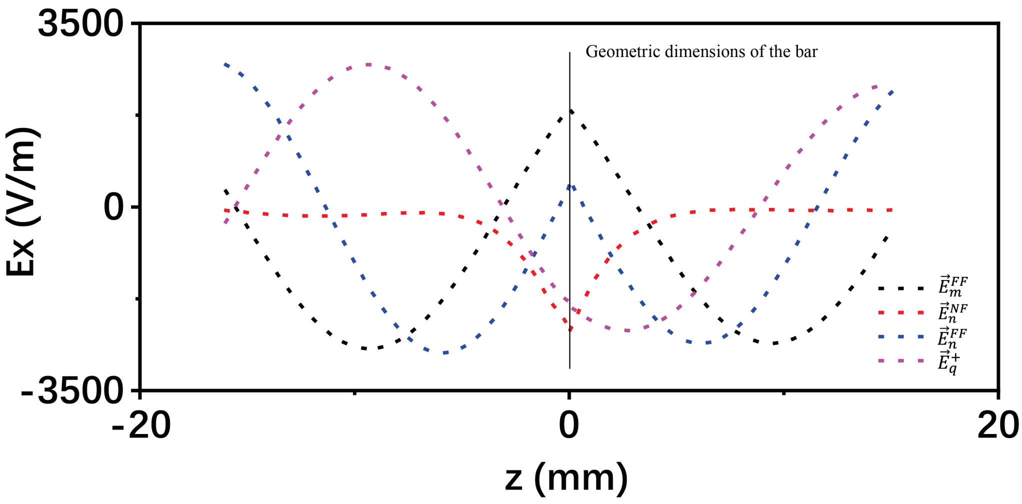


**Figure S1: Schematic diagram comparing the trends of the four electric field variations with the geometric sizes of the bar**

$\vec{E}_m^{\mathrm{FF}}$ refers to FF field distributions associated with the *m*-th resonator. $\vec{E}_n^{\mathrm{NF}}, \vec{E}_n^{\mathrm{FF}}$ refer to the NF and FF field distributions associated with the *n*-th resonator. $\vec{E}_q^{+}$ refers to the E fields of the incoming eigenmodes belonging to the *q*-th external port. As shown in Figure S1, $\vec{E}_m^{\mathrm{FF}}, \vec{E}_n^{\mathrm{NF}}, \vec{E}_n^{\mathrm{FF}}$and $\vec{E}_q^{+}$ these four electric fields are not the internal field of the resonator itself and vary very slowly with respect to the geometric dimensions of the bar. Therefore, in the integrals of Eq. (2) and Eq. (4), we can treat these four electric fields as constants perpendicular to the bar.

However, for $\langle \Psi_m^{\mathrm{NF}} | \Psi_m^{\mathrm{NF}} \rangle$, this cannot be handled in the same way, because the field involved is its own field, which does not change slowly with distance. It should be rewrited as:

$$\langle \Psi_m^{\mathrm{NF}} | \Psi_m^{\mathrm{NF}} \rangle = \frac{1}{2} \int d\tau \left[ \mu \vec{H}_m^{\mathrm{NF}*} \cdot \vec{H}_m^{\mathrm{NF}} + \varepsilon_\infty \vec{E}_m^{\mathrm{NF}*} \cdot \vec{E}_m^{\mathrm{NF}} + \omega_0^2 \left( \omega_p^2 \varepsilon_\infty \right)^{-1} \vec{P}_m^{*} \cdot \vec{P}_m + \left( \omega_p^2 \varepsilon_\infty \right)^{-1} \vec{J}_m^{*} \cdot \vec{J}_m \right]$$

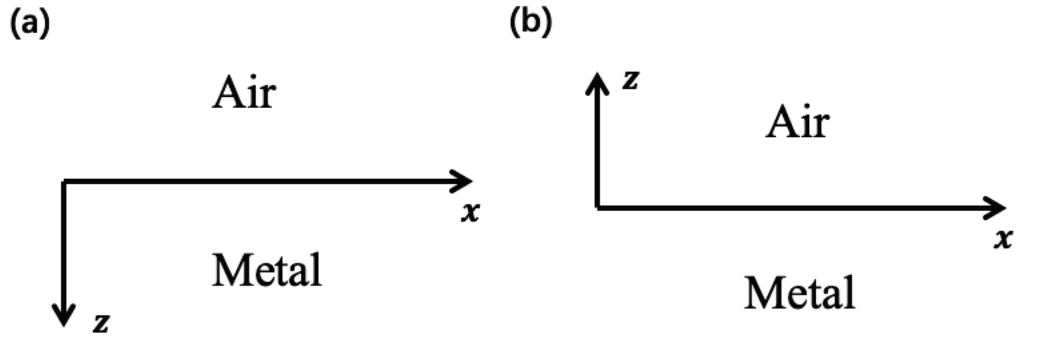


**Figure S2: SPP system: (a) in the metal, (b) in the air**

Now, let's consider an TM SPP system. When in metal, as shown in Figure S2a, according to Maxwell's equations, we can obtain:

$$\nabla\times\vec{H}=-i\omega\varepsilon\vec{E}\Rightarrow\vec{k}\times\vec{H}=-\omega\varepsilon\vec{E}\Rightarrow\begin{vmatrix} i & j & k\\ k_x & k_y & k_z\\ H_x & H_y & H_z\end{vmatrix}=-\omega\varepsilon\vec{E}\Rightarrow k_zH_y=\omega\varepsilon E_x\ ,\ k_xH_y=$$

$$\omega\varepsilon E_z\Rightarrow\left|\frac{k_z}{\omega\varepsilon}H_y\right|^2+\left|\frac{k_x}{\omega\varepsilon}H_y\right|^2=\left|\vec{E}\right|^2\Rightarrow\frac{\left|\vec{E}\right|^2}{\left|H_y\right|^2=\left|\vec{H}\right|^2}=\frac{|k_x|^2+\left|k_y\right|^2}{\omega^2|\varepsilon|^2}=\frac{k_x^2+k_x^2-n^2k_0^2}{\omega^2|\varepsilon|^2}=\frac{2k_x^2\left(1-\frac{\omega_p^2}{\omega^2}\right)k_0^2}{\omega^2\left(\frac{\omega_p^2}{\omega^2}\right)^2\varepsilon_0^2}\overset{(1)}{\Rightarrow}\approx$$

$$\frac{\frac{\omega_p^2}{\omega^2}k_0^2}{\omega^2\left(\frac{\omega_p^2}{\omega^2}\right)^2\varepsilon_0^2}=\frac{\frac{\omega_p^2}{\omega^2}*\left(\frac{\omega}{c}\right)^2}{\omega^2\left(\frac{\omega_p^2}{\omega^2}\right)^2\varepsilon_0^2}=\frac{\varepsilon_0\mu_0\omega^2}{\varepsilon_0^2\omega_p^2}=\frac{\mu_0\omega^2}{\varepsilon_0\omega_p^2}\Rightarrow\left|\vec{H}\right|^2=\left|\vec{E}\right|^2\frac{\varepsilon_0\omega_p^2}{\mu_0\omega^2}$$

When in air, as shown in Figure S2b, according to Maxwell's equations, we can obtain:

$$\nabla\times\vec{H}=-i\omega\varepsilon\vec{E}_0\Rightarrow\vec{k}\times\vec{H}=-\omega\varepsilon\vec{E}_0\Rightarrow\begin{vmatrix} i & j & k\\ k_x & k_y & k_z\\ H_x & H_y & H_z\end{vmatrix}=-\omega\varepsilon\vec{E}_0\Rightarrow k_zH_y,$$

$$k_xH_y=\omega\varepsilon E_{0z}\Rightarrow\left|\frac{k_z}{\omega\varepsilon}H_y\right|^2+\left|\frac{k_x}{\omega\varepsilon}H_y\right|^2=\left|\vec{E}\right|^2\Rightarrow\frac{\left|\vec{E}_0\right|^2}{\left|H_y\right|^2=\left|\vec{H}\right|^2}=\frac{|k_x|^2+\left|k_y\right|^2}{\omega^2|\varepsilon|^2}$$

$$=\frac{k_x^2+k_x^2-k_0^2}{\omega^2|\varepsilon|^2}=\frac{2k_x^2-k_0^2}{\omega^2\varepsilon_0^2}\overset{(1)}{\Rightarrow}\approx\frac{k_0^2}{\omega^2\varepsilon_0^2}=\left(\frac{\omega}{c}\right)^2\frac{1}{\omega^2\varepsilon_0^2}=\frac{\varepsilon_0\mu_0\omega^2}{\omega^2\varepsilon_0^2}=\frac{\mu_0}{\varepsilon_0}$$

$$\Rightarrow\left|\vec{H}\right|^2=\left|\vec{E}_0\right|^2\frac{\varepsilon_0}{\mu_0}\Rightarrow\left|\vec{E}\right|^2=\left|\vec{E}_0\right|^2\frac{\omega^2}{\omega_p^2}$$

And $\langle\Psi_m^{\mathrm{NF}}|\Psi_m^{\mathrm{NF}}\rangle$ can be written as two parts including inside metal resonantor and outside (air):

$$\langle\Psi_m^{\mathrm{NF}}|\Psi_m^{\mathrm{NF}}\rangle=<\psi_m^{NF}|\psi_m^{NF}>_{in}+<\psi_m^{NF}|\psi_m^{NF}>_{out}$$

where

$$<\psi_m^{NF}|\psi_m^{NF}>_{in}$$

$$=\frac{1}{2}\int d\tau\left[\mu\vec{H}_m^{\mathrm{NF}*}\cdot\vec{H}_m^{\mathrm{NF}}+\varepsilon_\infty\vec{E}_m^{\mathrm{NF}*}\cdot\vec{E}_m^{\mathrm{NF}}+\omega_0^2\left(\omega_p^2\varepsilon_\infty\right)^{-1}\vec{P}_m^*\cdot\vec{P}_m+\left(\omega_p^2\varepsilon_\infty\right)^{-1}\vec{J}_m^*\cdot\vec{J}_m\right]$$

$$=\frac{1}{2}\int d\tau\left[u_0\left|\vec{E}_m^{NF}\right|^2e^{-2\alpha z}\frac{\varepsilon_0}{\mu_0}\frac{\omega_p^2}{\omega^2}+\varepsilon_0\left|\vec{E}_m^{NF}\right|^2e^{-2\alpha z}+\left(\omega_p^2\varepsilon_0\right)^{-1}\frac{\varepsilon_0^2\omega_p^4}{\omega^2}\left|\vec{E}_m^{NF}\right|^2e^{-2\alpha z}\right]$$

$$=\frac{1}{2}\int ds\left[\frac{1}{2\alpha}\ \varepsilon_0\left|\vec{E}_0\right|^2\frac{\omega^2}{\omega_p^2}\ \frac{\omega_p^2}{\omega^2}+\frac{1}{2\alpha}\ \varepsilon_0\left|\vec{E}_0\right|^2\frac{\omega^2}{\omega_p^2}+\left(\omega_p^2\varepsilon_0\right)^{-1}\frac{\varepsilon_0^2\omega_p^4}{\omega^2}\frac{1}{2\alpha}\ \left|\vec{E}_0\right|^2\frac{\omega^2}{\omega_p^2}\right]$$

$$\overset{(3)}{\Rightarrow}=\frac{1}{2}\int ds\left[\frac{c}{2\omega_p}\ \varepsilon_0\left|\vec{E}_0\right|^2+\frac{c}{2\omega_p}\varepsilon_0\left|\vec{E}_0\right|^2\frac{\omega^2}{\omega_p^2}+\frac{c\varepsilon_0}{2\omega_p}\left|\vec{E}_0\right|^2\right]$$

$$=\frac{1}{2}\int ds\left|\vec{E}_0\right|^2\left[\frac{\varepsilon_0c}{\omega_p}\ +\frac{\varepsilon_0c\omega^2}{2\omega_p^3}\right]=\frac{\varepsilon_0c}{2}\int ds\left|\vec{E}_0\right|^2\left[\frac{2\omega_p^2+\omega^2}{2\omega_p^3}\right]$$

$$<\psi_m^{NF}|\psi_m^{NF}>_{out}=\frac{1}{2}\int d\tau\left[u\vec{H}_m^{NF*}\vec{H}_m^{NF}+\varepsilon_\infty\vec{E}_m^{NF*}\vec{E}_m^{NF}\right]$$

$$= \frac{1}{2}\int d\tau \left[u_0\left|\vec{E}_0\right|^2 e^{-2\alpha z}\frac{\varepsilon_0}{\mu_0} + \varepsilon_0\left|\vec{E}_0\right|^2 e^{-2\alpha z}\right] = \frac{1}{2}\int ds \left[\frac{1}{2\alpha}\,\varepsilon_0\left|\vec{E}_0\right|^2 + \frac{1}{2\alpha}\,\varepsilon_0\left|\vec{E}_0\right|^2\right]$$

$$\overset{(4)}{\Rightarrow} = \frac{1}{2}\int ds \left[\frac{c\omega_p}{\omega^2}\,\varepsilon_0\left|\vec{E}_0\right|^2\right] = \frac{\varepsilon_0 c}{2}\int ds\left|\vec{E}_0\right|^2\frac{\omega_p}{\omega^2}$$

Finally, we can get the ratio of the energy of inside metal resonator to the energy of outside (air):

$$\frac{<\psi_m^{NF}|\psi_m^{NF}>_{in}}{<\psi_m^{NF}|\psi_m^{NF}>_{out}} = \frac{2\omega_p^2+\omega^2}{2\omega_p^3} * \frac{\omega^2}{\omega_p} = \frac{2\omega_p^2\omega^2+\omega^4}{2\omega_p^3} = \frac{\omega^2}{\omega_p^2} + \frac{1}{2}\frac{\omega^4}{\omega_p^4}$$

Where $\omega$ refers to the frequency of the resonantor and $\omega_p$ is the plasma frequency, which is generally taken as $2\pi * 2.175 * 10^{15}s^{-1}$. And we can observe that when the frequency of the resonator decreases, the energy ratio becomes smaller.

At the same time, we also performed numerical calculations of this energy ratio for different frequency shown in following table:

**Table S1: Numerical calculations of energy ratio**

| Frequency | 224.35THz | 90.50THz | 0.53THz | 12GHz |
|---|---|---|---|---|
| $\frac{<\psi_m^{NF}\|\psi_m^{NF}>_{in}}{<\psi_m^{NF}\|\psi_m^{NF}>_{out}}$ | 0.1331 | 0.0712 | 0.0096 | 6.0145E-11 |

From the numerical calculations, we can also see that as the frequency decreases, the internal energy of the resonantor relative to the external becomes smaller and smaller. Thus, in the microwave frequency range, we can neglect the internal energy of the resonantor.

So when in the microwave frequency range

$$\langle\Psi_m^{\mathrm{NF}}|\Psi_m^{\mathrm{NF}}\rangle =< \psi_m^{NF}|\psi_m^{NF}>_{out}$$

**Some notes for this Section:**

(1) $k_x \approx k_0$

When $\omega \ll \frac{\omega_p}{\sqrt{2}}$, $k_x \approx k_0$ as shown in follow figure:

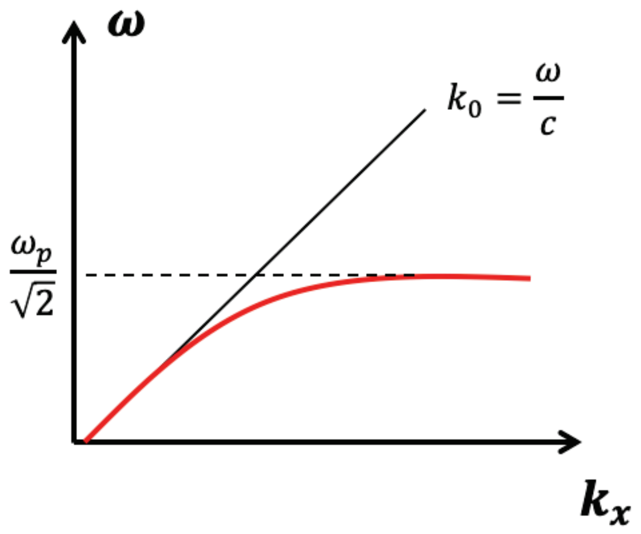


**Figure S3: The dispersion relation of SPP**

(2) For the SPP system, the parallel wave vector $k_{||}$ should satisfy:

$$k_{\backslash\backslash} = \frac{\omega}{c}\sqrt{\frac{\varepsilon_{air}\varepsilon_{metal}}{\varepsilon_{air} + \varepsilon_{metal}}}$$

(3) For a plane wave propagating along the z-direction, the exponential term can be written as (in metal):

$$e^{ik_z z} = e^{-\alpha z} \Longrightarrow ik_z = -\alpha \ \Longrightarrow \alpha = ik_z = -i\sqrt{k_0^2 - k_x^2} \ = -i * i\sqrt{k_x^2 - n^2 k_0^2}$$

$$\overset{(1)}{\Rightarrow} \approx \sqrt{\frac{\omega_p^2}{\omega^2} k_0^2} = \frac{\omega_p}{\omega} k_0 = \frac{\omega_p}{c}$$

(4) For a plane wave propagating along the z-direction, the exponential term can be written as (in air):

$$e^{ik_z z} = e^{-\alpha z} \Rightarrow ik_z = -\alpha \Rightarrow \alpha = ik_z = -i\sqrt{k_0^2 - k_x^2} = -i * i\sqrt{k_x^2 - k_0^2}$$

$$= \sqrt{k_x^2 - k_0^2} \overset{(2)}{\Rightarrow} = \sqrt{\frac{\omega^2}{c^2}\frac{\varepsilon}{1+\varepsilon} - k_0^2} = \sqrt{\frac{\omega^2}{c^2}\left(\frac{\varepsilon}{1+\varepsilon} - 1\right)} = \frac{\omega}{c}\sqrt{\left(\frac{-1}{1+\varepsilon}\right)}$$

$$= \frac{\omega}{c}\sqrt{\left(\frac{-1}{2 - \frac{\omega_p^2}{\omega^2}}\right)} \approx \frac{\omega}{c}\sqrt{\frac{\omega^2}{\omega_p^2}} = \frac{\omega^2}{c\omega_p}$$

## Section III – $E_x^{\mathrm{NF}}$ distribution of plasmonic resonators at optical frequency

The following figure shows the near field of the plasmonic resonators, where it can be seen that its near field can be well described using a dipole.

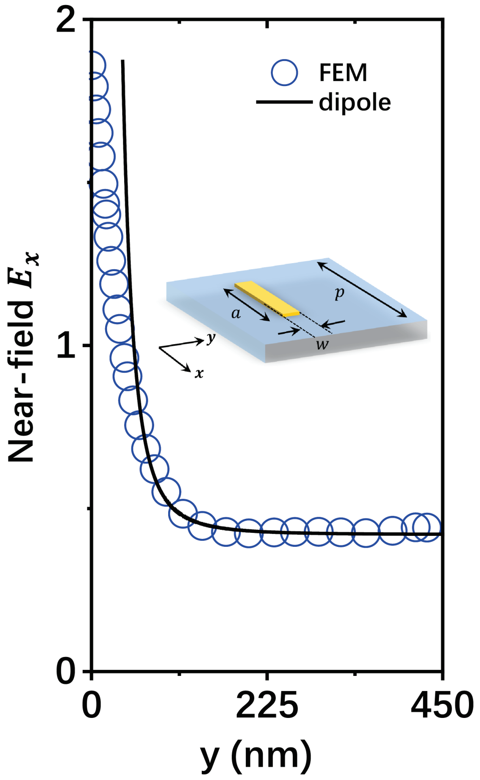


**Figure S4: FEM-simulated $E_x^{\mathrm{NF}}$ distribution of Plasmonic bar-resonator at optical frequency of 224THz as function of distance y along the centerline perpendicular to the resonator. Geometrical parameters: $p = 600nm, a = 300nm, w = 40nm$. And the thickness of metal and the dielectric are 45nm and 3um, respectively, and $\varepsilon_r$=2.4.**

Fig.S4 illustrate how the FEM simulated $E_x^{\mathrm{NF}}$ of a single bar-resonator varies along the centerline perpendicular to the bar at working frequency of 224GHz, which can be well described with a dipole model.

**Section IV – Derivation of Eq. (8)**

We diagonalize the matrix containing $t$ in Eq. (7) by an orthogonal transformation $\mathbf{M}$, i.e.

$$\mathbf{M}\begin{pmatrix}\omega_1 & t\\ t & \omega_2\end{pmatrix}\mathbf{M}^{-1}=\begin{pmatrix}\widetilde{\omega}_+ & 0\\ 0 & \widetilde{\omega}_-\end{pmatrix}$$

$$\mathbf{M}\begin{pmatrix}a_1\\ a_2\end{pmatrix}=\begin{pmatrix}\tilde{a}_+\\ \tilde{a}_-\end{pmatrix}$$

$$\mathbf{M}\begin{pmatrix}-\Gamma_1 & X\\ X & -\Gamma_2\end{pmatrix}\mathbf{M}^{-1}=\begin{pmatrix}-\tilde{\Gamma}_+ & \tilde{X}\\ \tilde{X} & -\tilde{\Gamma}_-\end{pmatrix}$$

$$\mathbf{M}\begin{pmatrix}\kappa_{11}\\ \kappa_{21}\end{pmatrix}=\begin{pmatrix}\tilde{\kappa}_{11}\\ \tilde{\kappa}_{21}\end{pmatrix}$$

And $\mathbf{M}$ can be expressed by

$$\mathbf{M}=\begin{pmatrix}\dfrac{\Delta\omega+\sqrt{\Delta\omega^2+t^2}}{\sqrt{t^2+\left(\Delta\omega+\sqrt{\Delta\omega^2+t^2}\right)^2}} & \dfrac{t}{\sqrt{t^2+\left(\Delta\omega+\sqrt{\Delta\omega^2+t^2}\right)^2}}\\ \dfrac{\Delta\omega-\sqrt{\Delta\omega^2+t^2}}{\sqrt{t^2+\left(\Delta\omega-\sqrt{\Delta\omega^2+t^2}\right)^2}} & \dfrac{t}{\sqrt{t^2+\left(\Delta\omega-\sqrt{\Delta\omega^2+t^2}\right)^2}}\end{pmatrix}$$

## Section V – Physical mechanism of the BIC for the system #3 in Fig. 4

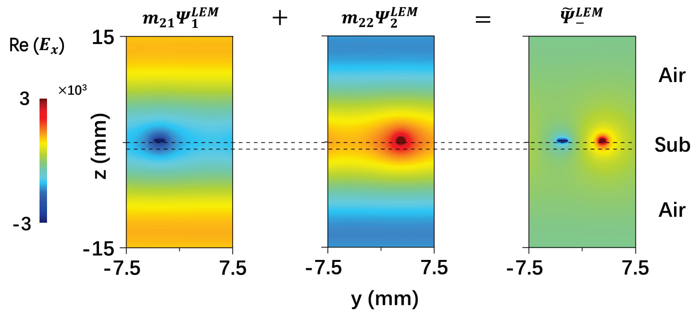


**Figure S5: Physical mechanism of the BIC. FEM-computed-field distributions of the system containing the wider resonator only in the left panel, the system containing the thinner resonator only in the middle panel, and the whole system in the right panel, at the BIC frequency for #3.**

Here, we present the FEM-computed Ex-field distribution of the two individual bar-resonators and the entire whole system #3 at the BIC working frequency in Fig. S7 in SM. The wave-function of the "dressed" mode is a linear combination of two original modes[1], $\tilde{\Psi}_-^{\rm LEM} = m_{21}\Psi_1^{\rm LEM} + m_{22}\Psi_2^{\rm LEM}$, with $m_{21}$ and $m_{22}$ being two coefficients in orthogonal transformation M (i.e., the same matrix diagonalizing equation (7)). Tuning the value of $t$ can dramatically alter the coefficients $m_{21}$ and $m_{22}$ (see more details in Sec. IV of SM), thereby modifying the total radiation of the "dressed" mode. At the BIC frequency, we find $t =$ 1.13 and thus $m_{21}$ =-0.65and $m_{22}$ =0.76, causing the radiations from two original modes to completely cancel each other (ses Fig. S7), leading to the formation of the BIC.

## Section VI – Details information about single resonators in Figs. 4 and 5

For the first five points marked with #1-5 in Fig. 4, the constraints are given in the main text, specifically. And the structural parameters are given in the main text, with the values of being 7.4mm, 6.8mm, 5.6mm, 4.4mm, and 3.4mm, respectively.

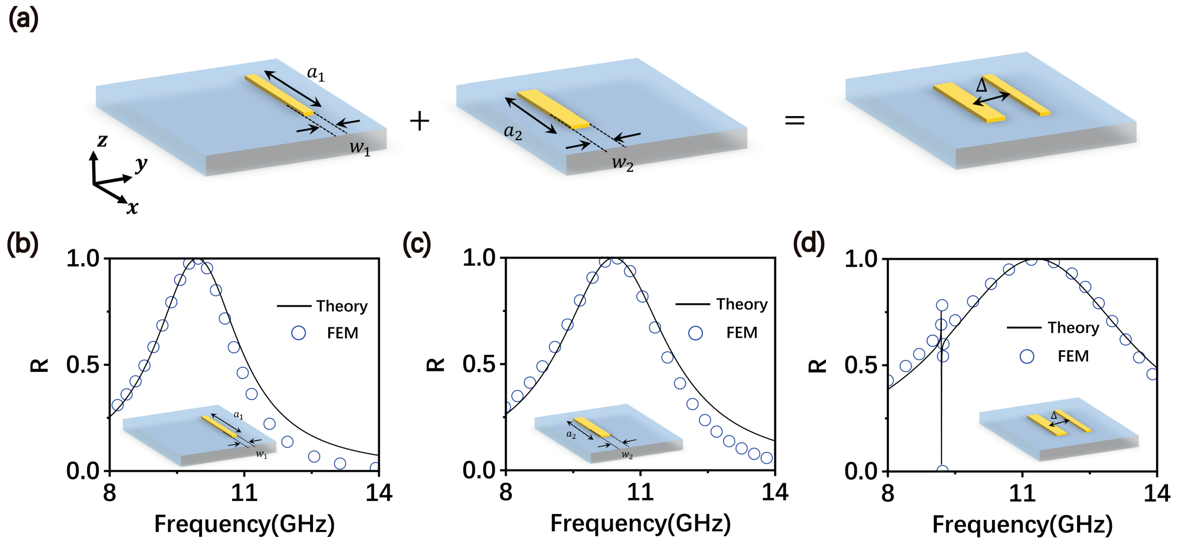


**Figure S6: (a) Schematics of realistic design with coupled metasurfaces based on two coupled bar-resonators for system #4 Shown in Fig. 4. Geometrical parameters:$a_1 = 12\text{mm}, w_1 = 0.45\text{mm}, a_2 = 11.5\text{mm}, w_2 = 1.25\text{mm}$, and the period is 15mm. All metallic resonators of the fabricated metasurfaces are made of copper with thickness of 35 um and placed on a dielectric substrate (with $\varepsilon_r$=2.2) with thickness of 1 mm. the distance between two bar-resonator is 4.4mm. (b-d) Reflectance spectra of designed periodic metasurfaces containing different single bar-resonator (b-c) and two bar-resonators coupled together (d), obtained by FEM simulation (circles) and LEM theory (solid line).**

As mentioned in the main text, the system #1-#5 are of the original optical properties with $\Delta\omega = 0.35, \Delta\Gamma = 0.33$. Here is the meta-atom design and EM response which can satisfy the above-mentioned condition. Figure S5 shows the theoretically computed and FEM-simulated reflection spectra of two individual bar-resonator (b-c) and the coupled system #4 based on two coupled resonators (d) mentioned in the main text. FEM simulations (circles) agree very well with LEM Theory (solid line) results.

Figure S6 illustrate the realistic design of the system #7 in Fig. 4 with $\Delta\omega = 3.04, \Delta\Gamma = 0.33$. To have better control on the near-field coupling strength and meet the condition of ($\Delta\omega = 3.04$), we need more space to adjust the relative positions of the two resonators, thus we use C-shape resonators as meta-atom design as shown in Figure S6. Figure S6 shows the theoretically computed and FEM-simulated reflection spectra of two individual bar-resonator (b-c) and the coupled system #7 based on two coupled resonators (d) mentioned in the main text. FEM simulations (circles) agree very well with LEM Theory (solid line) results.

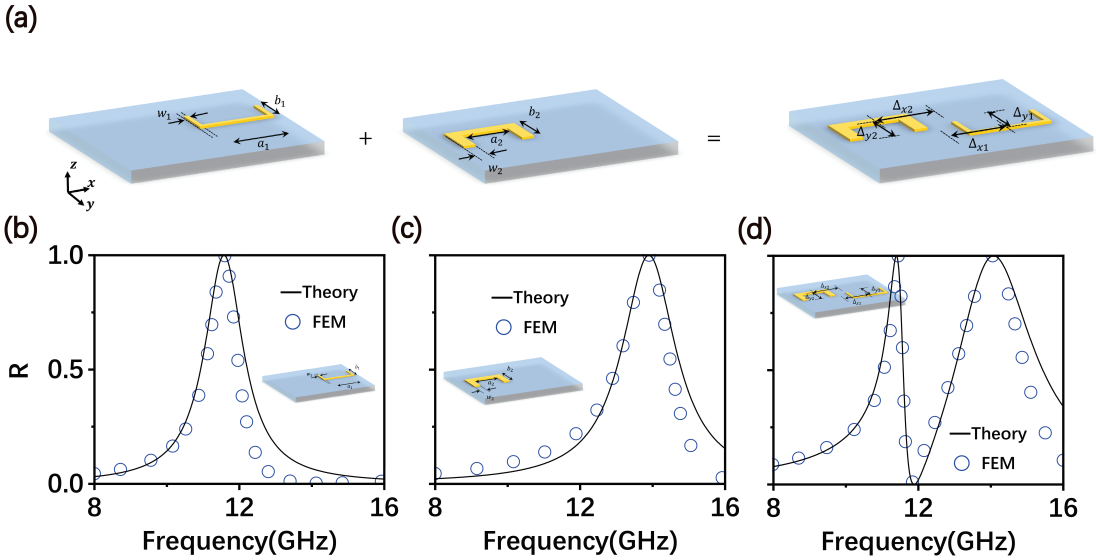


**Figure S7: (a) Schematics of realistic design with coupled metasurfaces based on two coupled resonators for system #7 shown in Fig5. Geometrical parameters: :$a_1 = 5.08$mm, $w_1 = 0.4$mm, $b_1 = 2.93$mm, $a_2 = 3.5$mm, $w_2 = 1.3$mm, $b_2 = 2.15$mm, and the period is 15mm. All metallic resonators of the fabricated metasurfaces are made of copper with thickness of 35 um and placed on a dielectric substrate (with $\varepsilon_r$=2.2) with thickness of 1 mm. (b-d) Reflectance spectra of designed periodic metasurfaces containing different single resonator (b-c) and two resonators coupled together (d), obtained by FEM simulation (circles) and LEM theory (solid line).**

**Table S2: Additional geometrical parameter for the Meta-systems shown in Fig. 5**

| Serial number / parameters (mm) | 6 | 7 | 8 | 9 | 10 |
|---|---|---|---|---|---|
| $\Delta_{x1}$ | 0 | 0 | 4 | 4 | 4 |
| $\Delta_{y1}$ | 0.5 | 0.5 | 0 | 0 | 0 |
| $\Delta_{x2}$ | 0 | 0 | -3.6 | -3.6 | -3.6 |
| $\Delta_{y2}$ | -5.5 | -3.5 | -2.7 | -1 | 0 |

Table S2 shows the specific parameters of the structure in Fig. S6, where $\Delta_x$ and $\Delta_y$ represent the coordinate positions of the structure's center, respectively.

## Section VII – Reflection spectrum experimental system

**Under the condition of normal incidence in the experiment, the reflection spectrum is not easy to measure directly. Simply put, our experimental setup consists of two horn antennas and a sample, as shown in Fig. S8. During the testing, in fact, there is a small incident angle between the horn antenna and the sample, approximately 5°. This is an issue that cannot be avoided in the experiment.**

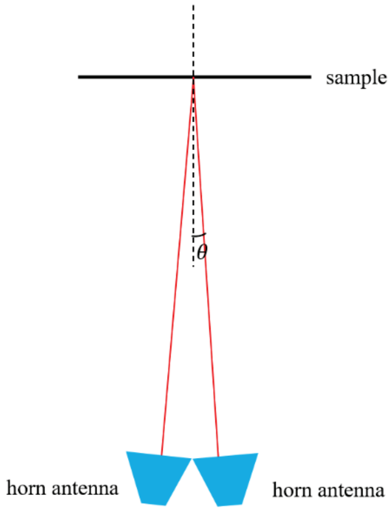


**Figure S8: Schematic diagram of the reflection spectrum experimental testing system**

**Section VIII – Applicability of LEM theory for oblique incident case**

**Our extended LEM theory can apply to oblique incident case, provided that the meta-atoms comprising the metasurface exhibit subwavelength characteristics. To examine this further, we conducted both FEM simulations and LEM-based calculations to re-investigate the electromagnetic response of the meta-system shown in Fig. 2d under normal and oblique incidence for both TE and TM polarizations. As shown in Fig. S9 a-c, for TM polarization, there is excellent agreement between the LEM calculations and FEM simulations for normal incidence and for oblique incidence at 5°, but a noticeable discrepancy arises at 20° incidence. In contrast, for TE polarization, good agreement persists even at 20° oblique incidence.**

**The underlying physics can be attributed to polarization dependence of the incident wave vector $k_0$. For TM polarized wave, the wave vector $k_0$ has a component along the x-direction (see Fig. S9e), which effectively increases the unit cell size along this direction at higher incidence angles. This degrades the subwavelength nature of the metasurface and allows undesired modes to appear near the frequency range of interest, thereby violating the single-mode approximation required by the LEM theory, as discussed in Ref. [1].**

**As a result, the mode wavefunctions become hybridized, leading to inaccuracies in the coupling parameters in Eq. (2) of the main text, and ultimately causing the discrepancies observed in Fig. S9c. On the other hand, for TE polarization, $k_0$ does not have a component along the x-direction, so the subwavelength condition remains intact and the calculations remain accurate, as shown in Fig. S9d.**

**To further validate this explanation, we designed a metasurface based on "H"-shaped meta-atoms with improved subwavelength performance for TM-polarized waves. As shown in Figs. S9g and h, excellent agreement is achieved between the LEM-calculated and FEM-simulated results under both normal and 20° oblique incidence, confirming the robustness of our theoretical framework under the appropriate subwavelength conditions.**

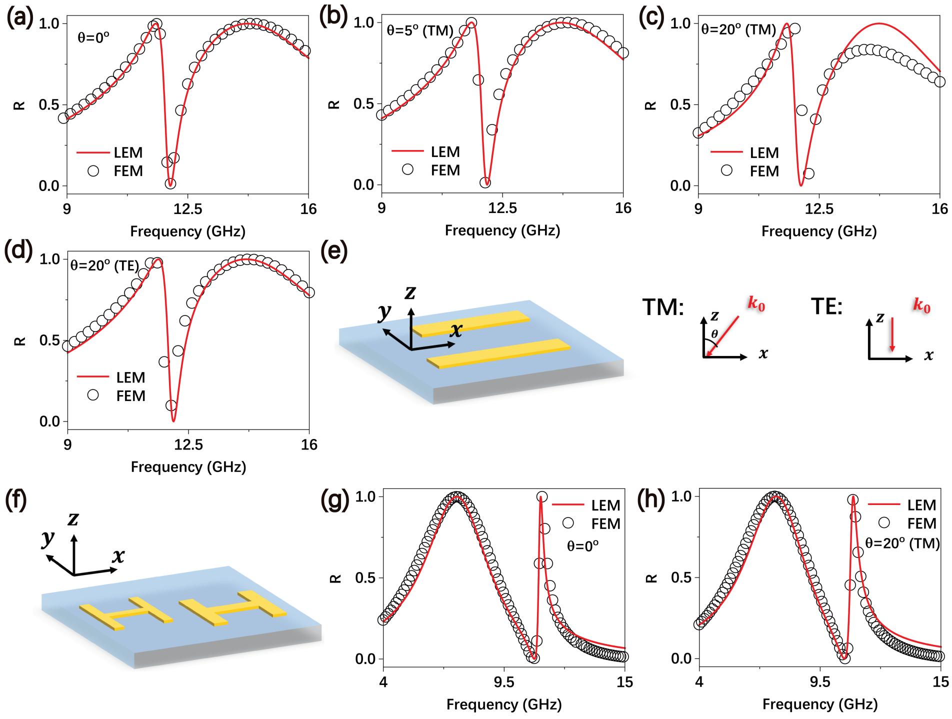


**Figure S9. Verification of oblique incidence: (a-d) Reflectance spectra of the bar system under oblique incidence; (e) shows the schematic of the structure and the distribution of the wave vector $k_0$ under oblique incidence; (f) represents the "H"-shaped system with better subwavelength properties; (g-h) show the reflectance spectra of the "H"-shaped system under oblique incidence.**

## References


[1] J. Lin *et al.*, "Tailoring the lineshapes of coupled plasmonic systems based on a theory derived from first principles," *Light Sci. Appl.*, vol. 9, p. 158, 2020.

[2] A. Raman and S. Fan, "Photonic Band Structure of Dispersive Metamaterials Formulated as a Hermitian Eigenvalue Problem," *Phys. Rev. Lett.*, vol. 104, no. 8, p. 087401, 2010.
